# Human CD34^+^-derived complete plasmacytoid and conventional dendritic cell vaccine effectively induces antigen-specific CD8^+^ T cell and NK cell responses in vitro and in vivo

**DOI:** 10.1007/s00018-023-04923-4

**Published:** 2023-09-20

**Authors:** Jesper van Eck van der Sluijs, Diede van Ens, Jolanda Brummelman, Daan Heister, Aastha Sareen, Lisa Truijen, Dorette S. van Ingen Schenau, Mirjam H. M. Heemskerk, Marieke Griffioen, Michel G. D. Kester, Nicolaas P. M. Schaap, Joop H. Jansen, Anniek B. van der Waart, Harry Dolstra, Willemijn Hobo

**Affiliations:** 1 Department of Laboratory Medicine, Laboratory of Hematology, Radboud University Medical Center, Radboud Institute for Molecular Life Sciences, Geert Grooteplein 8, P.O. Box 9101, 6500 HB Nijmegen, The Netherlands; 2 Princess Maxima Center for Pediatric Oncology, Utrecht, The Netherlands; 3 Department of Hematology, Leiden University Medical Center, Leiden, The Netherlands; 4 Department of Hematology, Radboud University Medical Center, Nijmegen, The Netherlands

**Keywords:** CD34^+^ progenitor cells, Immunotherapy, Dendritic cells, Vaccination, T cells, NK cells

## Abstract

Allogeneic stem cell transplantation (alloSCT) can be curative for hemato-oncology patients due to effective graft-versus-tumor immunity. However, relapse remains the major cause of treatment failure, emphasizing the need for adjuvant immunotherapies. In this regard, post-transplantation dendritic cell (DC) vaccination is a highly interesting strategy to boost graft-versus-tumor responses. Previously, we developed a clinically applicable protocol for simultaneous large-scale generation of end-stage blood DC subsets from donor-derived CD34^+^ stem cells, including conventional type 1 and 2 DCs (cDC1s and cDC2s), and plasmacytoid DCs (pDCs). In addition, the total cultured end-product (DC-complete vaccine), also contains non-end-stage-DCs (i.e. non-DCs). In this study, we aimed to dissect the phenotypic identity of these non-DCs and their potential immune modulatory functions on the potency of cDCs and pDCs in stimulating tumor-reactive CD8^+^ T and NK cell responses, in order to obtain rationale for clinical translation of our DC-complete vaccine. The non-DC compartment was heterogeneous and comprised of myeloid progenitors and (immature) granulocyte- and monocyte-like cells. Importantly, non-DCs potentiated toll-like receptor-induced DC maturation, as reflected by increased expression of co-stimulatory molecules and enhanced cDC-derived IL-12 and pDC-derived IFN-α production. Additionally, antigen-specific CD8^+^ T cells effectively expanded upon DC-complete vaccination in vitro and in vivo*.* This effect was strongly augmented by non-DCs in an antigen-independent manner. Moreover, non-DCs did not impair in vitro DC-mediated NK cell activation, degranulation nor cytotoxicity. Notably, in vivo i.p. DC-complete vaccination activated i.v. injected NK cells. Together, these data demonstrate that the non-DC compartment potentiates DC-mediated activation and expansion of antigen-specific CD8^+^ T cells and do not impair NK cell responses in vitro and in vivo*.* This underscores the rationale for further clinical translation of our CD34^+^-derived DC-complete vaccine in hemato-oncology patients post alloSCT.

**Supplementary Information:**

The online version contains supplementary material available at 10.1007/s00018-023-04923-4.

## Introduction

Despite the powerful T and natural killer (NK) cell-mediated graft-versus-tumor (GVT) immune responses post allogeneic stem cell transplantation (alloSCT), relapse remains the main cause of treatment failure for hemato-oncology patients [1, 2]. This may, amongst others, be attributed to insufficient presentation of tumor-associated antigens (TAAs), neo-antigens or minor histocompatibility antigens (MiHA) by antigen-presenting cells (APC). It may also result from suboptimal activation, expansion and functionality of tumor-reactive T and NK cells [3]. This highlights the medical need for adjuvant therapeutic strategies post-alloSCT. Approaches that boost graft-versus-tumor immunity may hold the key to establishing long-lasting tumor control.

In this perspective, dendritic cell (DC) vaccination is highly attractive, as DCs are the most professional APCs and powerful orchestrators of innate and adaptive immunity. However, the clinical efficacy of monocyte-derived DC (moDC) vaccination in cancer patients has been limited so far. Alternatively, naturally occurring blood DC subsets are postulated to be more powerful and their use in vaccination approaches is actively pursued [4, 5]. The human DC compartment in blood is generally divided in conventional type 1 DCs (cDC1s, CLEC9A^+^CD141^+^), conventional type 2 DCs (cDC2s, CD1c^+^CD11c^+^) and plasmacytoid DCs (pDCs, CD123^+^CD303^+^) [6, 7]. Numerous (pre-)clinical studies demonstrated that these DC subsets are critical tailors of anti-tumor T and NK cell responses. As established in cDC1- and cDC2-deficient mouse models, the cross-presenting cDC1s are initiators of CD8^+^ T cell-mediated tumor control [4, 8, 9]. In contrast, cDC2s mainly orchestrate CD4^+^ T cell-mediated anti-tumor immunity [10, 11]. Importantly, presence of cDC1s or cDC2s in tumors correlated with a better overall survival in a variety of human cancers [8, 12]. Next, pDCs are classically recognized as type I interferon (IFN)-producing cells in response to viral infection, however, they also orchestrate anti-tumor T and NK cell responses [13, 14]. For example, pDC vaccination in B16 melanoma- or acute lymphoblastic leukemia-bearing mice resulted in NK cell-mediated tumor control [15, 16]. More importantly, accumulating data demonstrated that (in)direct cross-talk between cDC1s, cDC2s and pDCs led to robust and powerful induction of innate and adaptive immune responses. cDC1s and cDC2s collaborate to maximize the activation of CD8^+^ T cells via a two-step activation process, involving CD4^+^ T cell help. cDC2-activated CD4^+^ T cells license cDC1s via CD40-CD40L interaction (step 1), thereby augmenting cDC1’s ability to prime and activate CD8^+^ T cells (step 2)[17, 18]. Furthermore, cDC1s were demonstrated to actively recruit pDCs to the cDC1-CD8^+^ T cell priming site, whereupon pDC-derived type I IFN boosted the immune priming capacity of cDC1s [19–21]. In addition, targeted antigen-delivery to pDCs resulted in pDC-derived exosome-mediated antigen transfer to cDC1s. In turn, these cDC1s robustly induced antigen-specific CD8^+^ T cell responses [22]. Interestingly, the DC cross-talk has also been observed in cancer patients, where intradermal co-injection of CpG-B (for pDC activation) and GM-CSF (for cDC activation) resulted in enhanced recruitment of cDC1s to the sentinel lymph node via a type I IFN-dependent mechanism. Consequently, reduced metastatic spread was observed in melanoma patients [23].

Together, this supports the clinical attractiveness of a pan-DC vaccine, containing cDC1s, cDC2s and pDCs. Their unique functional features and cross-talk capacity are anticipated to broadly boost GVT immunity and improve tumor control post-alloSCT. Due to limited numbers of DCs in the blood, especially cDC1s (< 0.05% of leukocytes), other sources with easier access that offer sufficient cell numbers for multiple vaccinations are explored. In this perspective, CD34^+^ hematopoietic progenitor cells (HPC) provide a unique opportunity for ex vivo generation of naturally occurring blood DC subsets, especially in the context of alloSCT for high-risk hemato-oncology patients. Therefore, we have developed a clinically applicable manufacturing process for large scale generation of highly functional naturally occurring cDC1s, cDC2s, and pDCs from a small fraction of a donor CD34^+^ stem cell graft [24]. Vaccination with the total cultured end-product (i.e. DC-complete vaccine) would be most ideal to avoid additional time-consuming and expensive isolation procedures (i.e. sorting procedures). However, besides the end-stage blood DC subsets, the DC-complete vaccine also contains other cell types, i.e., non-end-stage-DCs (further referred to as non-DCs). To allow eventual clinical translation of this DC-complete vaccine, we aimed to first unravel the phenotypic identity of the non-DCs and their potential immune modulatory functions on cDC and pDC potency in stimulating tumor-reactive CD8^+^ T cell and NK cell responses in vitro and in vivo.

## Materials and methods

### Patient and donor material

CD34^+^ HPCs were obtained from leukapheresis material of G-CSF mobilized healthy donors. For in vitro and in vivo cytomegalovirus (CMV)-specific T cell assays, autologous CD3^+^ T cells containing low frequencies of CMV-specific CD8^+^ T cells were used. For in vitro MiHA-specific T cell expansion assays, we used cryopreserved peripheral blood mononuclear cells (PBMCs) containing HA-1-specific CD8^+^ T cells, obtained post-alloSCT. NK cells were freshly isolated from donor buffy coats obtained from Sanquin Blood Supply Foundation. Patient characteristics are described in Table S1. All patient material was obtained in accordance with the Declaration of Helsinki and institutional guidelines and regulations (CMO 2013/064).

### Ex vivo generation of CD34^+^ HPC-derived DC-based vaccine

**Fig. 1:**
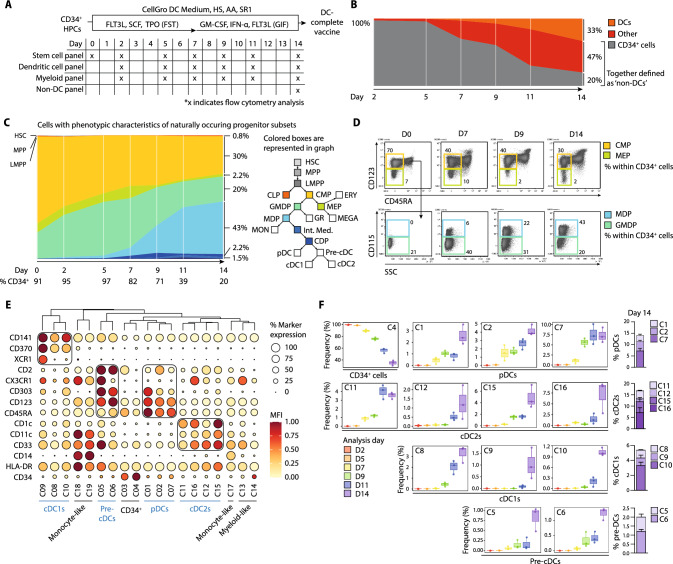
Dynamic development of CMP, GMDP, MDP into CDP and end-stage DC subsets. **a** Schematic overview of our culture system, including specification of the flow cytometry panels used to characterize the developmental process. All experiments were performed for three independent donors (*n* = 3). **b** Fish plot (mean percentage) visualizing the culture dynamics over time. **c** Fish plot (mean percentage) and **d** representative flow cytometry plots displaying differentiation within the CD34^+^ progenitor cell compartment over time by manual gating of the stem cell panel (Fig. S2). Colors indicated in the hematopoietic tree represent the colors in the fish plot. **e** Balloon plot displaying marker expression (percentage positivity and MFI of positive cells) within 19 hierarchically ordered FlowSOM metaclusters of the DC panel. **f** Frequency of annotated metaclusters obtained with the DC panel, over time, presented in box plots. Total frequency per DC subset at the end of culture is displayed in a stacked histogram. Data is shown as mean ± SEM. *DC* dendritic cell; non-DC, non-end-stage DC, *HSC* Hematopoietic Stem Cell, *MPP* Multipotent Progenitor, *LMPP* Lymphoid-Myeloid Primed Progenitor, *CLP* Common Lymphoid Progenitor, *CMP* Common Myeloid Progenitor, *MEP* Megakaryocyte Erythrocyte Progenitor, *GMDP* Granulocyte Monocyte Dendritic cell Progenitor, *MDP* Monocyte Dendritic Cell Restricted Progenitor, *CDP* Common Dendritic Cell Progenitor, *MON* monocytes, *GR* granulocytes, *MFI* median fluorescence intensity

The CD34^+^ HPC-derived DC-based vaccine products were generated as described previously [24]. Briefly, MACS purified CD34^+^ (≥ 90%) HPCs were cultured for 14 days in CellGenix® GMP DC medium supplemented with 2% human serum (HS, Sanquin Blood Supply Foundation), 1 µM StemReginin1 (SR1, Cellagen Technology) and 50 µg/mL ascorbic acid (AA, Sigma Aldrich). From day 0–7, medium was supplemented with 100 ng/mL FLT3L, SCF and TPO (all ImmunoTools). From day 7–14, medium was supplemented with 800 IU/mL GM-CSF (ImmunoTools), 1000 IU/mL IFN-α (Roche) and 100 ng/mL FLT3L. For phenotypic evaluation either 1 × 10^5^ (day 0), 2 × 10^5^ (day 2, 5) or 1 × 10^6^ (day 7, 9, 11, 14) cells were used per respective flow cytometry (FCM) staining (Fig. 1a). For functional assays, either the total cultured end product was directly used (DC-complete vaccine), or DC subsets and non-DCs were individually sorted (Fig S1) using the Aria II SORP sorter (BD Bioscience). The pan-DC vaccine was generated by re-mixing sorted cDC1s, cDC2s and pDCs at the same ratios as in the total cultured end-product (Fig. 3a).

### Flow cytometry

Cells were resuspended in 50 µL brilliant stain buffer (BD Biosciences) supplemented with 0.1 mg/mL total human immunoglobulins (Sanquin Blood Supply Foundation) and incubated for 10 min at 4 °C. Antibody mixtures were prepared in 0.5% human serum albumin (HSA, Sanquin blood Supply Foundation)/PBS buffer, centrifuged for 2 min at 14,000*g* and 50 µL was added to cells. Cells were vortexed and incubated for 20 min at 4 °C in the dark, then washed twice and resuspended in 0.5% HSA/PBS buffer and analyzed on the CytoFLEX LX flow cytometer (Beckman Coulter). Other FCM stainings were performed as described previously [24]. Absolute counts in in vivo experiments were performed using flow count fluorospheres (Beckman Coulter). Cells were analyzed on CytoFLEX LX or Gallios flow cytometers (both Beckman Coulter). FCM panels are depicted in Table S2.

### High-dimensional flow cytometry data analysis

Flow Cytometry Standard (FCS) 3.0 files were pre-processed in Kaluza (v2.1). Compensation was performed, followed by FSC/SSC gating and exclusion of doublets, dead cells and fluorochrome aggregates. For each panel, relevant cell populations (for the DC panel all viable CD19^−^ cells and for the non-DC panel all viable cells) were exported to CSV files and imported in R (v4.0.5), whereupon data was analyzed as described by Brummelman et al*.* [25]. In brief, data was arcsine transformed using the transFlowVS function of the flowVS package (v1.19.0) and scaled and centered using the scale function. Multi-dimensional scaling (MDS) plots, based on the median expression values of each marker, were generated using the plotMDS function from the limma package (v3.44.3). Next, all samples were down sampled to 40,000 events, concatenated and cell subpopulations were identified using the FlowSOM algorithm [26]. The optimal number of metaclusters was defined as 19 and 20 for the DC panel and the non-DC panel, respectively. Heatmaps were created using the pheatmap package (v1.0.12), where hierarchical clustering was performed using the Euclidean distance and Ward-linkage. Additional figures were made using the ggplot2 package (v3.3.5), e.g. balloonplots. Cluster data were embedded in the FCS files, which were then further analyzed in Flowjo (v.9) to evaluate metacluster phenotype by exporting the frequencies of positive cells and corresponding median fluorescence intensity (MFI). Clusters defined by non-specific staining and debris were excluded from analysis. Fish plots were generated using the Fishplot *R* package (v0.5.1)[27]. Scaling within MST and balloon plots was performed per marker. This results in loss of relative expression identification to other antigens (which is highly dependent on fluorochrome choice), but ensures identification of relative differences between clusters. Manual verification, as applied in the balloon plots, takes actual positivity (%) and spreading errors into account in contrast to a clustering algorithm that visualizes non-specific staining as expression.

### Maturation and cytokine release assays

Enriched DC subsets (25 × 10^3^), the pan-DC vaccine (25 × 10^3^ DCs at the same ratios as in the respective DC-complete vaccine) and the DC-complete vaccine (cell count adjusted per culture to contain 25 × 10^3^ DCs) were resuspended in IMDM/10% FCS/1% Pen/Strep (PS, Life Technologies) and supplemented with 10 ng/mL IL-3 (ImmunoTools) and  U/mL GM-CSF, seeded in 96-wells round bottom plates. For overnight maturation, either 0.5 µM CpG-P (TLR-9 agonist, Miltenyi Biotec), 5 µg/mL R848 (TLR-7/8 agonist, Enzo Lifesciences) + 20 µg/mL Poly I:C (TLR-3 agonist, Sigma Aldrich) (abbreviated as RPI:C) or a combination of 0.5 µM CpG-P + 20 µg/mL RPI:C (P-RPI:C) was added. Cell culture supernatants were used to investigate IL-12p70 (eBiosciences) and IFN-α (MabTech) cytokine release by ELISA according to manufacturer’s instructions. Phenotypic maturation was examined by FCM on CytoFLEX (Beckman Coulter), subsequently integrated MFIs (iMFIs) (% marker positive multiplied by MFI of positive cells) and balloon plots were generated.

### In vitro T cell expansion assays

For CMV assays, the DC-based vaccines were generated from HLA-A2^+^ alloSCT-donors having CMV-reactive CD8^+^ memory T cells. For MiHA assays, the DC vaccines were generated from HLA-A2^+^ HA-1^−^ alloSCT-donors. Sorted DC subsets, non-DCs, pan-DC vaccine and DC-complete vaccine were overnight matured/stimulated using CpG-P, RPI:C or the combination (P-RPI:C) and, when indicated, loaded with 5 µM short-peptide (CMV: NLVPMVATV or HA-1: VLHDDLLEA, both LUMC-IHB peptide facility). Next, DC subsets, pan-DC vaccine and DC-complete vaccines (all containing 0.5 × 10^5^ end-stage DCs), were seeded in 24-wells flat bottom plates. Cells were co-cultured with 5 × 10^5^ autologous MicroBead isolated CD3^+^ T cells in IMDM/10% FCS/1% PS (Pan T cell enrichment kit, Miltenyi Biotec, CMV assays) or 7.5 × 10^5^ patient-derived PBMCs (MiHA assays) in IMDM/10% HS/1% PS. For CMV assays, 0.25–1.5 × 10^6^ non-DCs were added resulting in ratios of 1:0.5, 1:1 and 1:3 DC:non-DCs, where the 1.5 × 10^6^ non-DCs mimicks the situation in the DC-complete vaccine. This 1:3 ratio was therefore used in the MiHA and transwell (Corning, 0.4 µM pore size) assays. For transwell assays, T cells were labeled with carboxylfluorescein succinimidyl ester (CFSE, ThermoFisher). Unstimulated total CD8^+^ T cells were used as a negative control for CFSE-dilution. Cells were incubated for 7 days. At day 5, half of the medium was refreshed and supplemented with 50 IU/mL IL-2 (Chiron) and 5 ng/mL IL-15 (ImmunoTools). At day 7, cells were harvested, counted using trypan blue exclusion and tetramer^+^ antigen-specific CD8^+^ T cell frequencies were analyzed on a Gallios flow cytometer (Beckman Coulter).

### In vitro NK cell assays

Sorted DC subsets, pan-DC vaccine and DC-complete vaccine (all containing 0.5 × 10^5^ end-stage DCs) were seeded in 96-wells round bottom plates in IMDM/10% FCS/1% PS. Next, DC subsets and pan-DC vaccine were co-cultured with/without 0.5 × 10^5^ or 1 × 10^5^ non-DCs (1:1 and 1:2 ratio DC:non-DC, respectively). Cells were matured/stimulated using P-RPI:C for 2 h followed by washing. Then, 0.5 × 10^5^ healthy donor NK cells, isolated using EasySep™ NK cell isolation kit (Stem Cell Technologies), were added and cells were co-cultured for 40 h (DC:NK ratio of 1:1). Subsequently, cell culture supernatants were harvested for IL-12p70, IFN-α and IFN-γ (MabTech) cytokine release using ELISA. Moreover, phenotypic NK cell activation was determined by flow cytometry [24]. Additionally, a 4 h killing and degranulation assay was performed by adding 2.5 × 10^4^ CFSE labelled THP-1 cells and anti-CD107a PECy7 to the culture (BioLegend). Finally, viable THP-1 cells were quantified on a CytoFLEX flow cytometer (without washing). The percentage of killing was determined as follows: 100—((absolute number of viable CFSE^+^ THP-1 cells co-cultured with NK cells/absolute number of viable CFSE^+^ THP-1 cells cultured without NK cells) × 100). THP-1 phenotype was validated using flow cytometry.

### In vivo studies

Animal experiments were approved by the Dutch Central Committee of Animal Experiments (CCD 2015–0120 and 2018–0029) and the Animal Experimental Committee of the Radboudumc. Experiments were performed in accordance with national and institutional guidelines and compliant with relevant legislation under the university permit number 10300. All in vivo experiments complied with the ARRIVE guidelines. Mice were inspected daily on general behavior, and weight was monitored every 2–3 days. In all experiments we used female immune-deficient NOD/SCID/IL2Rγ^null^ (NSG) mice (Jackson Laboratories, in-house breeding) at 6–20 weeks of age. A random number generator was used to randomize treatments across the animals prior to start of experiment. This was performed within each cage to eliminate cage effects and age or litter induced differences. All researchers involved were blinded for treatment during the experiment. De-blinding was performed after completion of analyses.

For in vivo T cell expansion assays 32 mice (model 1) and 16 mice (model 2) at *n* = 8 per group were used (Fig. 5a, d). Mice received intravenous (i.v.) infusion of human peripheral blood lymphocytes (PBLs) containing CMV-specific CD8^+^ T cells. Model 1: 20 × 10^6^ PBLs containing ~ 50,000 CMV-specific (1.0% of CD8^+^) T cells. Model 2: 12.5 × 10^6^ PBLs containing ~ 37,500 CMV-specific (0.8% of CD8^+^) T cells. Mice were treated at day 0 and 7 with i.p. injections containing 0.5 × 10^6^ mature peptide-loaded end-stage DCs (i.e. pDCs, cDC1s or cDC2s only, the pan-DC vaccine or the DC-complete vaccine). To support human T cell engraftment, 0.5 µg rhIL-15 (ImmunoTools) was administered i.p. every 2–3 days. At days 7 and 14, blood was analyzed for the presence of CMV-specific CD8^+^ T cells by flow cytometry.

For the in vivo NK cell expansion assay 42 mice, *n* = 7 per group were used. Mice received i.v. infusion of 3 × 10^6^ human NK cells, followed by i.p. vaccination with DC-complete vaccine containing 0.5 × 10^6^ mature end-stage DCs. At days 0, 2 and 5, mice received 0.5 µg or 2.5 µg rhIL-15 i.p. to support human NK cell engraftment, or PBS as control. At days 1 and 4 blood was collected. At day 7, blood, spleen, bone marrow and liver were harvested and analyzed for NK cell frequencies and activation state. For the in vivo tumor model 51 mice, *n* = 17 per group were used. Mice sacrificed because of non-experimental related humane endpoints were censored from data analyses, resulting in *n* = 14–16 mice per treatment condition. THP-1 cells encoding a luciferase GFP reporter system (LucGFP THP-1, validated phenotype using flow cytometry) [28] were used for longitudinal tumor-load monitoring using bioluminescence imaging (BLI). At days − 1 or 0, mice received intrafemoral (i.f.) infusions of 2 × 10^4^ LucGFP THP-1 cells in one femur. Subsequently, at days 1 and 6, mice received i.v. infusion with 3 × 10^6^ healthy donor NK cells from the 1 donor (9 NK cell donors were used in total, equally distributed over both NK cell treated groups) or PBS as control. In parallel, part of the isolated NK cells from each donor were cultured in IMDM + 10% HS supplemented with low-dose IL-15 (0.3 ng/mL) until second infusion. At days 1, 3, 6 and 8, mice were i.p. treated with DC-complete vaccine containing 0.5 × 10^6^ DCs or PBS as control. To support NK cell survival, 0.5 µg rhIL-15 or PBS was injected i.p. every 2–3 days until day 28. At days 2, 4, 7 and 9 blood was analyzed by flow cytometry to investigate NK cell activation (*n* = 11 per analysis timepoint due to animal welfare restrictions in maximum blood volume withdrawal). Tumor load was monitored every week by BLI starting from day 6 onwards, prior to second NK cell infusion, as previously described [29]. Metastases in the non-injected femur, belly (high above reproduction organs, including liver and spleen) and/or reproductive organs was scored by two independent researchers through blind analyses of the BLI data. Mice were closely monitored on weight, general appearance, behavior and the development of a palpable tumor. Mice were sacrificed using cervical dislocation according to well-defined humane endpoints or at the end of the experiment (day 84). Humane endpoints included: a palpable tumor > 1cm^3^, paralysis, > 15% weight loss in two days, > 20% weight loss compared to the start of the experiment and general appearance (fur, skin, reactivity, posture and/or pain).

Blood and bone marrow were processed using BD Pharm Lyse™ to lyse erythrocytes. Single cell suspensions of spleen and liver were processed by density-based separation using Lympholyte (SanBio). Cells were analyzed using flow cytometry, either for tetramer staining and memory differentiation (T cells) or activation profile (NK cells). Absolute counts were performed by stain-lyse-no-wash method with the addition of Flow-count Fluorospheres (Beckman Coulter).

### Statistical analyses

Statistical analyses were performed, as indicated, using GraphPad Prism (v5.0.3). P-values < 0.05% were considered statistically significant (***P < 0.001, **P < 0.01, *P < *0.05)*. For all mice experiments, power calculation for primary outcome was performed using estimations for effect size and data variation based on existing data of in vivo antigen-specific CD8^+^ T cell expansion and NK activation and anti-tumor effect in similar models and approved by the Animal Welfare Body (Radboud University) after consultation with a statistician.

## Results

### The non-DC compartment is heterogenous and contains CD34^+^ progenitor cells, (immature) monocyte- and granulocyte-like cells

To dissect the dynamics of CD34^+^ progenitor differentiation into DC subsets in our 2-week culture process [24], we performed longitudinal high-dimensional flow cytometry. Three different 14–20 marker panels (Table S2) were used to extensively characterize hematopoietic progenitor cells, monocytic/granulocytic differentiation and DC differentiation (Figs. 1a; S2 and S3). We observed that the proportion of CD34^+^ progenitor cells gradually decreased over time to an average of 20% at day 14 (Fig. 1b, DC panel). At day 0, the culture predominantly consisted of cells with phenotypic characteristics of naturally occurring common myeloid progenitors (CMPs), megakaryocyte erythrocyte progenitors (MEPs) and granulocyte macrophage dendritic cell progenitors (GMDPs) (Figs. 1c–d, S3, Stem cell panel). Cells with the phenotypic characteristics of hematopoietic stem cells (HSCs), multipotent progenitors (MPPs), lympho-myeloid primed progenitors (LMPPs) and common lymphoid progenitors (CLPs) accounted for < 1% of the CD34^+^ cells. Over time, a relative decrease was observed in phenotypically identified CMPs, MEPs, and GMDPs. Notably, this was coincided by an increase in lineage-committed monocyte dendritic progenitors (MDPs) and common dendritic progenitors (CDPs), and the appearance of end-stage DC subsets (Fig. 1c–d). By applying automated clustering analysis (FlowSOM) to the data obtained with our DC panel, we defined 19 metaclusters (Fig. 1e). We identified 2 clusters enriched for progenitor cells (CD34^+^), 2 pre-cDC clusters, 10 DC subset clusters and 5 clusters consisting of monocytes (CD14^+^CD33^+^) and other cells of myeloid origin (CD14^−^CD33^+^). Importantly, over time, multiple pDC, cDC2 and cDC1 subsets could be detected (Figs. 1f, S2c). Earliest cDC2s and pDCs were detectable at day 7, including the CD45RA^bright^ pDCs (cluster 1). The potentially more differentiated CX3CR1^+^ cDC2s (cluster 16) increased in proportion from day 11 onwards. cDC1s (clusters 8, 9, 10) could be detected from day 9 onwards.

The populations with higher CD141 expression (cluster 10), and CD370 and XCR1 expression (cluster 9) arose later compared to cluster 8, hinting at sequential development and differentiation of the early-developed cDC1s. In total, the culture consisted on average of 14.1% pDC (range 11.4–17.8%), 5.4% cDC1 (range 3.8–6.5%) and 16.8% cDC2 (range 14.7–18.6%) at the end of the culture (Fig. 1f).

**Fig. 2:**
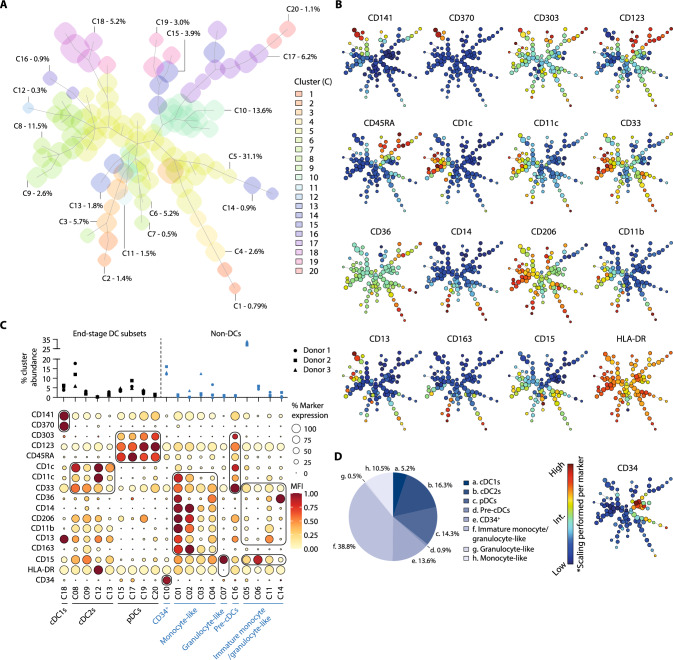
The non-DC compartment is comprised of a heterogenous mixture of (immature) granulocyte-, monocyte- and myeloid-like cells. The non-DC panel was used to investigate the non-DC fraction in three independent donors (*n* = 3) **a** Minimal spanning tree (MST) showing 100 nodes with allocated metaclusters (shaded background) and average abundance at day 14 of the culture system. **b** Relative expression of each marker within every FlowSOM node plotted on the MST. Low expression does not equal marker negativity, scaling was performed per marker. **c** Balloon plot representing marker expression (percentage positivity and MFI of positive cells) within the 20 FlowSOM metaclusters, including cell type annotation (non-DCs in blue) and relative abundance per donor. **d** Pie chart visualizing the composition of the DC-complete vaccine (mean of each group of FlowSOM metaclusters). *MFI* median fluorescence intensity, *DC* dendritic cell, *cDC* conventional dendritic cell, *pDC* plasmacytoid dendritic cell

In order to eventually clinically translate the DC-complete vaccine, it is crucial to characterize the non-DC compartment in further detail. Therefore, we applied a more extensive myeloid phenotyping panel combining monocytic/granulocytic markers with DC subset markers (Fig. S4, Table S2, non-DC panel). Using the FlowSOM algorithm we defined 20 metaclusters, represented in a minimal spanning tree (MST) of 100 nodes (Fig. 2a, b). Here, we again identified our DC subsets (clusters 8, 9, 12, 13, 15, 17–20), pre-cDCs (cluster 16; CD123^bright^CD33^+^CD303^+−^) and CD34^+^ cells (cluster 10) (Fig. 2c). The remaining non-DCs contained monocyte-like cells (10%, clusters 1–4), granulocyte-like cells (0.5%, cluster 7) and immature monocyte/granulocyte-like cells (39%, clusters 5, 6, 11 and 14) (Fig. 2c, d). The monocyte-like clusters had varying co-expression signatures (percentage and MFI level) of CD36, CD11b, CD33, CD13 and the scavenger receptors CD206 and CD163. These monocyte-like cells expressed CD11c similar to the cDCs, but also expressed monocyte lineage marker CD14, while lacking end-stage DC markers. Within the immature cell populations, cluster 11 resembled the CD14^+^ monocyte-like clusters, with CD11b and CD13 expression and partial expression of CD14, CD36 and CD206. Cluster 14 exhibited elevated expression of CD36, while lacking CD14 and CD11b, suggesting the presence of monoblast-like cells. Cluster 6 may represent (pro)myelocytes (CD15^bright^), as these cells had lower HLA-DR and CD141 expression and more closely resembled the CD11b^−^ granulocyte-like cells. Finally, cluster 5 was comprised of a relatively large portion of cells (30%) which expressed CD33 and HLA-DR, but lacked marker expression of mature myeloid cells.

Together, by mapping the dynamic process of CD34^+^ HPC-to-DC development we revealed ongoing differentiation and expansion of natural DC subsets. The non-DC compartment consisted of CD34^+^ progenitor cells and (immature) monocyte- and granulocyte-like cells at various differentiation stages, which allows ongoing development of additional antigen-presenting cells.

### Non-DC have no negative effect on pan-TLR induced functional maturation of pDCs and cDCs

**Fig. 3:**
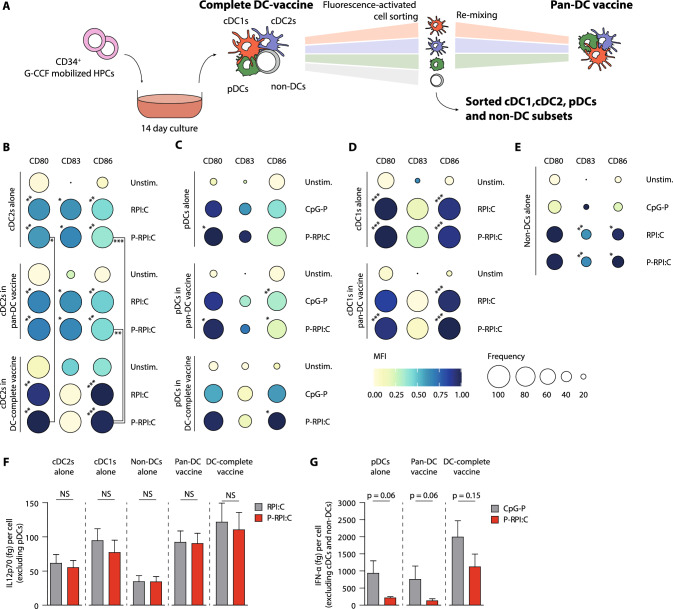
Non-DC have no negative effect on pan-TLR induced functional maturation of cDCs and pDCs. **a** Schematic overview visualizing the different vaccine strategies, including the DC-complete vaccine, sorted cDC1, cDC2, pDC and non-DC vaccines, and the Pan-DC vaccine which is generated by re-mixing cDC1s, cDC2s and pDCs according to the natural composition in the DC-complete vaccine. All experiments were performed with 3 independent donors. **b–e** Expression of co-stimulatory molecules CD80, CD83 and CD86 on unstimulated, RPI:C or CpG-P stimulated and P-RPI:C stimulated cDC2s **b**, pDCs **(c)**, cDC1s **d** and non-DCs **e** as represented in balloon plots showing the frequency of positive cells (size of the balloon) and the MFI (color intensity). Asterisks at the upper left corner of the balloons represent statistically significant differences in iMFI of TLR stimulated cells versus unstimulated cells. Asterisks below indicated lines represent statistically significant differences for P-RPI:C stimulated cells within the different vaccine strategies per respective end-stage DC subset. Data is shown as mean ± SEM. **f–g** Release of pro-inflammatory cytokines IL-12p70 per potential IL-12p70 producing cell (total µg produced / total number of cells excluding pDCs) **f** and IFN-α per IFN-α producing cell (total µg produced / number of pDCs) **g** upon RPI:C/CpG-P and P-RPI:C stimulation of the different vaccine strategies. Data is shown as mean ± SEM. Statistical analyses were performed using repeated measures one-way ANOVA followed by Bonferroni correction comparing all pairs of columns **b–e** or using a non-parametric paired T-test **f–g**. *P < 0.05, **P < 0.01, ***P < *0.001.* iMFI, integrated median fluorescence intensity; P-RPI:C, CpG-P + R848 + Poly I:C; cDC, conventional dendritic cell; pDC, plasmacytoid dendritic cell; non-DCs, non-end-stage dendritic cells; MFI, median fluorescence intensity; SEM, standard error of the mean

As the non-DC compartment contains amongst others (immature) monocytes with potential antigen-presentation capacity, it is important to determine whether they exert immunomodulatory effects within the total DC-complete vaccine. Therefore, we evaluated the impact of non-DCs presence on end-stage-DC subset maturation and DC’s potency to stimulate T and NK cells. Hence, cDC1s, cDC2s, pDCs and non-DCs were sorted from the DC-complete vaccine to obtain the individual subset vaccines (Fig. 3a and S1). Next, Pan-DC vaccines were generated by re-mixing cDC1s, cDC2s and pDCs according to the their natural composition in the DC-complete vaccine (Fig. 3a). First, we compared individual (CpG-P or RPI:C) versus the pan TLR ligand P-RPI:C maturation/stimulation cocktails for DC subset maturation in the presence/absence of non-DCs. Upon TLR stimulation, cDC2s, pDCs and cDC1s strongly upregulated expression of co-stimulatory molecules CD80, CD83 and CD86, as demonstrated by significantly increased iMFIs (Figs. 3a–c, S5a–c). Notably, combined TLR-stimulation (P-RPI:C) induced similar expression of co-stimulatory molecules by each subset compared to individual CpG-P or RPI:C stimulation (Figs. 3a–c, S5a–c). Interestingly, also the non-DC fraction upregulated the expression of these co-stimulatory molecules upon RPI:C and P-RPI:C stimulation (Figs. 3d, S5d), which may be indicative of APC features. Unfortunately, cDC1 maturation could not be evaluated in the DC-complete vaccine after TLR-stimulation due to upregulation of CD141 on non-DCs. Most importantly, in the presence of non-DCs, cDC2s demonstrated significantly higher expression of CD80 and CD86 compared to cDC2s alone or cDC2s in the pan-DC vaccine (P-RPI:C stimulation, Figs. 3a, S5e).

Next, we investigated the release of pro-inflammatory cytokines IL-12p70 by cDCs and IFN-α by pDCs upon TLR-stimulation. Inclusion of CpG-P in the TLR-stimulation did not alter the IL-12p70 production capacity of cDCs (Figs. 3e, S4f-h). Interestingly, the non-DC population also produced IL-12p70 (Fig. 3e), but not IFN-α upon TLR activation (Fig. S5g). Although P-RPI:C stimulated pDCs seemed to produce lower levels of IFN-α compared to CpG-P stimulated pDCs, this was not significant (Figs. 3f, S4g–h). Most importantly, high IL-12p70 and IFN-α levels were observed in the TLR-activated DC-complete vaccine.

To summarize, P-RPI:C stimulation effectively induced simultaneous phenotypic and functional maturation of pDCs, cDCs and (a subfraction of) the non-DC compartment. Moreover, presence of non-DCs potentiated TLR-mediated maturation of cDCs, as reflected by increased expression of co-stimulatory molecules CD80 and CD86 on cDC2s and high production of IL-12p70 and IFN-α.

### Non-DCs strongly potentiate in vitro DC-mediated antigen-specific CD8^+^ T cell expansion in an antigen-independent manner

Tumor-reactive CD8^+^ T cell responses play a key role in the GVT response post-alloSCT [30]. We previously demonstrated that CD34^+^ HPC-derived DC subsets potently boost the expansion of tumor-reactive CD8^+^ T cells [24]. Here, we investigated the potential impact of non-DC presence on DC-mediated T cell activation and assessed whether the DC-complete vaccine could effectively induce antigen-specific CD8^+^ T cell expansion.

**Fig. 4:**
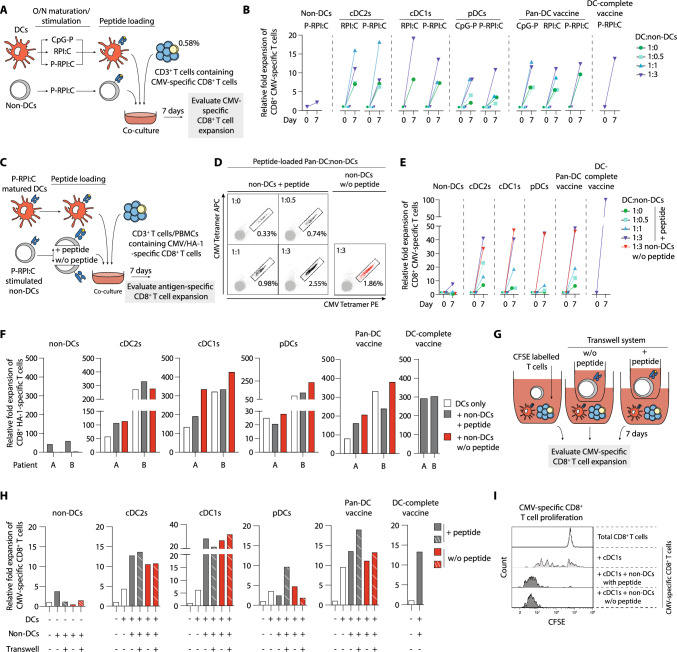
Non-DC strongly augmented DC-mediated antigen-specific T cell expansion in an antigen-independent manner. Assays were performed for 5 different experiments to assess CMV- (*n* = 3; **b**, **e**, **h**, **i**) or HA1-specific (*n* = 2; **f**) memory CD8^+^ T cell expansion potential of the different vaccination strategies. **a** Schematic overview of CMV-specific CD8^+^ T cell expansion upon stimulation with mature peptide-pulsed DC in the presence/absence of TLR-stimulated non-DCs pulsed with peptide. **b** Fold expansion of CMV-specific CD8^+^ T cells upon 7 day co-culture. Representative data of one independent donor is shown. **c** Schematic overview of antigen-specific CD8^+^ T cell expansion upon stimulation with mature peptide-pulsed DC in the presence/absence of TLR-stimulated non-DCs pulsed with/without peptide. **d–f** Representative CMV-tetramer flow cytometry plots **d** and relative fold expansion of CMV-specific CD8^+^ T cells of a second independent donor **e** and of HA-1-specific CD8^+^ T cells of 2 independent patients **f**. HA-1 assay: the DC:non-DC ratio was equal to the natural composition in the DC-complete vaccine. **g** Schematic overview of the transwell assay to examine the impact of (in)direct presence of non-DCs on DC-mediated CMV-specific CD8^+^ T cell expansion. **h** Fold expansion of CMV-specific CD8^+^ T cells upon mature peptide-pulsed DC stimulation in the presence of direct (full bars) or indirect exposure to TLR-stimulated non-DCs (striped bars). **i** Representative histogram of a third independent donor showing CMV-specific CD8^+^ T proliferation upon stimulation with cDC1s in the absence/presence of (non)-peptide-pulsed non-DCs. Unstimulated total CD8 + T cells were used as a negative control for CFSE-dilution. *DC* dendritic cell, *cDC* conventional dendritic cell, *pDC* plasmacytoid dendritic cell, *non-DC* non-end-stage dendritic cell, P-RPI:C, CpG-P + R848 + Poly I:C, *CMV* cytomegalovirus, *CFSE* carboxyfluorescein succinimidyl ester

TLR-activated and CMV peptide-pulsed DC subsets in the presence/absence of non-DCs were co-cultured with autologous CD3^+^ T cells containing low frequencies of CMV-specific CD8^+^ T cells for 7 days, whereupon CMV-specific CD8^+^ T cell expansion was assessed (Fig. 4a). First, we observed that non-DCs alone already induced moderate CMV-specific CD8^+^ T cell expansion. In addition, the sorted DC subsets and the pan-DC vaccine effectively stimulated expansion of CMV-specific CD8^+^ T cells, which was strongly augmented by the presence of non-DCs in a dose-dependent manner (Fig. 4b). Accordingly, the DC-complete vaccine also robustly boosted the expansion of CMV-specific CD8^+^ T cells. Notably, no differences were observed in fold expansion between CpG-P, RPI:C or P-RPI:C matured end-stage-DCs and therefore all subsequent experiments were only performed using P-RPI:C maturation (Fig. 4b).

Next, we examined whether the stimulatory effect of non-DCs was dependent on its antigen presentation capacity (Fig. 4c). Interestingly, even without peptide pulsing, non-DCs continued to enhance DC-mediated antigen-specific CD8^+^ T cell expansion (Fig. 4d–h). Notably, the DC-complete vaccine, which naturally contains non-DCs, exhibited a profound ability to amplify the expansion of CMV-specific CD8^+^ T cells (Fig. 4e). Furthermore, we confirmed that non-DCs also potentiated DC-mediated expansion of tumor-reactive (MiHA) CD8^+^ T cells in a clinically relevant context using patient-derived HA-1-specific CD8^+^ T cells of two independent patients (Fig. 4f). To further investigate the mechanism underlying the stimulatory effect of non-DCs (e.g. via cell-to-cell contact or soluble factors), we employed a transwell assay (Fig. 4g). Remarkably, in either scenario, the non-DCs consistently augmented the DC-induced expansion of CMV-specific CD8^+^ T cells (Fig. 4h, i). These findings indicate that the beneficial effect of non-DCs is likely mediated through soluble factors, independent of cell–cell contact. In conclusion, in both the viral and the tumor-specific model, we demonstrated the potent ability of non-DCs to enhance the T cell stimulatory capacity of pDC and cDC subsets.

### In vivo vaccination with CD34^+^-derived DCs effectively induced expansion of antigen-specific CD8^+^ T cells

**Fig. 5:**
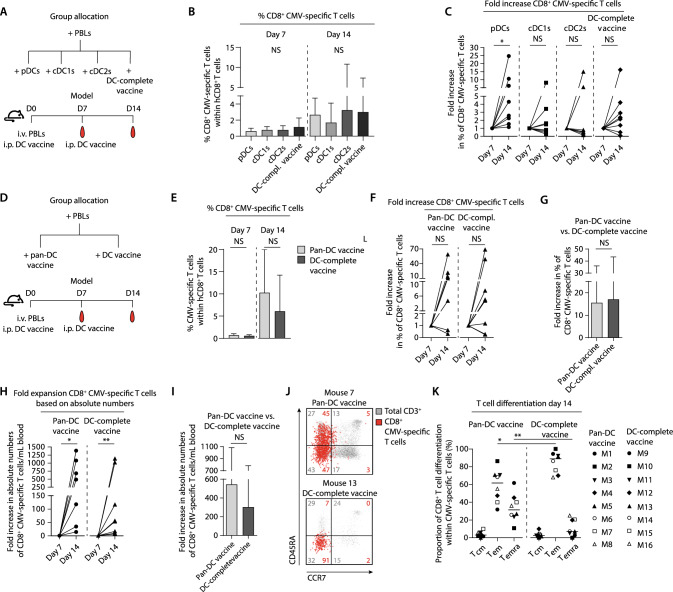
In vivo vaccination with CD34^+^-derived DCs effectively induces expansion of antigen-specific CD8^+^ T cells. **a**, **d** Schematic overview of group allocation and experimental setup of in vivo model 1 and model 2. PBLs containing CMV-specific CD8^+^ T cells were i.v. injected at day 0 (model 1: 20 × 10^6^ PBLs, containing ~ 50,000 CMV-specific CD8^+^ T cells, 1.0% of CD8^+^ T cells, *n* = 8 mice per group; model 2: 12.5 × 10^6^ PBLs, containing ~ 37,500 CMV-specific CD8^+^ T cells, 0.8% of CD8^+^ T cells, *n* = 8 mice per group), followed by treatment with vaccines containing 0.5 × 10^6^ mature peptide-pulsed end-stage DCs at day 0 and 7. **b**, **e** Frequencies of CMV-specific CD8^+^ T cells within human CD8^+^ T cells at day 7 and 14 after DC vaccination (*mean* ± *SD)*. **c**, **f–g** Fold increase of % CMV-specific CD8^+^ T cells and **h**, **i** absolute numbers of CMV-specific CD8^+^ T cells upon DC vaccination. **j** Representative dot plots and **k** combined data of T cell differentiation status (T_cm (_CD45RA^−^CCR7^+^), T_em (_CD45RA^−^CCR7^−^) and T_emra_ (CD45RA^+^CCR7^−^)) upon pan-DC and DC-complete vaccination. Numbers in the FACS plots indicate the percentage T_n_, T_cm_, T_em_ and T_emra_ within the CMV-specific CD8^+^ T cells. Statistical analysis was performed using a Kruskal–Wallis test followed by Dunn’s multiple comparisons test **b**, non-parametric paired T-test **c**, **f**, **h**, non-parametric unpaired T-test **e**, **g**, **j**, only comparing pan-DC versus DC-complete vaccine for all memory T cell subsets **j**. *P < 0.05, **P < *0.01.* *PBL* peripheral blood lymphocytes, *DC* dendritic cell, *cDC* conventional dendritic cell, *pDC* plasmacytoid dendritic cell, *i.v.* intravenous, *i.p.* intraperitoneally, *CMV* cytomegalovirus, *NS* non-significant, *T*_*cm*_ central memory T cells, *T*_*em*_ effector memory T cells, *T*_*emra*_ terminally differentiated effector memory T cells. to initiate tumor cell killing (Figs. 6c–d. S7a-c). Notably, the inclusion of cDCs/pDCs further enhanced the NK cell degranulation capacity and effectively induced NK cell-mediated tumor reactivity. Correspondingly, the DC-complete vaccine induced effective phenotypic and functional NK cell activation (Fig. 6b–d)

To further unravel the functional impact of non-DC presence, we next studied the in vivo T cell stimulatory capacity of the DC-complete vaccine compared to individual DC subset vaccines and the pan-DC vaccine. Hereto, PBLs containing low frequencies of CMV-specific CD8^+^ T cells were i.v. injected in NSG mice at day 0, followed by two i.p. infusions with mature CMV-peptide-pulsed DCs at days 0 and 7 (Fig. 5a, d, S6a–c, f–h). Functionality of the infused DCs was confirmed in vitro (S6a–c, f–h). Non-DC presence did not impair the engraftment of human CD45^+^ cells, CD3^+^ T cells nor CD8^+^ T cells (Fig. S6d-e, i-m). No statistically significant differences were observed between the DC vaccination strategies (Fig. 5b). Interestingly, between days 7 and 14, 8/8 pDC vaccinated mice exhibited an increase in CMV-specific CD8^+^ T cells compared to 6/8 DC-complete vaccine treated mice, 4/8 cDC1 vaccinated mice and only 2/8 cDC2 vaccinated mice (Fig. 5c). Importantly, both pan-DC and DC-complete vaccinated mice demonstrated increased CMV-specific CD8^+^ T cell frequencies (6/8 mice, Fig. 5e). No statistically significant differences in fold increase of percentage or absolute numbers of antigen-specific CD8^+^ T cells were observed between the Pan-DC and DC-complete vaccination strategies (Fig. 5f-i). Interestingly, the activated and expanded CMV-specific CD8^+^ T cells in the DC-complete vaccine exhibited a dominant effector memory T cell (T_em_) phenotype, whereas CMV-specific CD8^+^ T cells of pan-DC vaccinated mice showed substantial terminal differentiation (T_emra_) (Fig. 5j–k). Taken together, these data indicate that our CD34^+^-derived DC subsets, as well as the DC-complete vaccine, potently boosted the expansion of antigen-specific CD8^+^ T cells in vivo.

### DC-complete vaccination results in strong induction of NK cell activation and cytolytic capacity in vitro

**Fig. 6:**
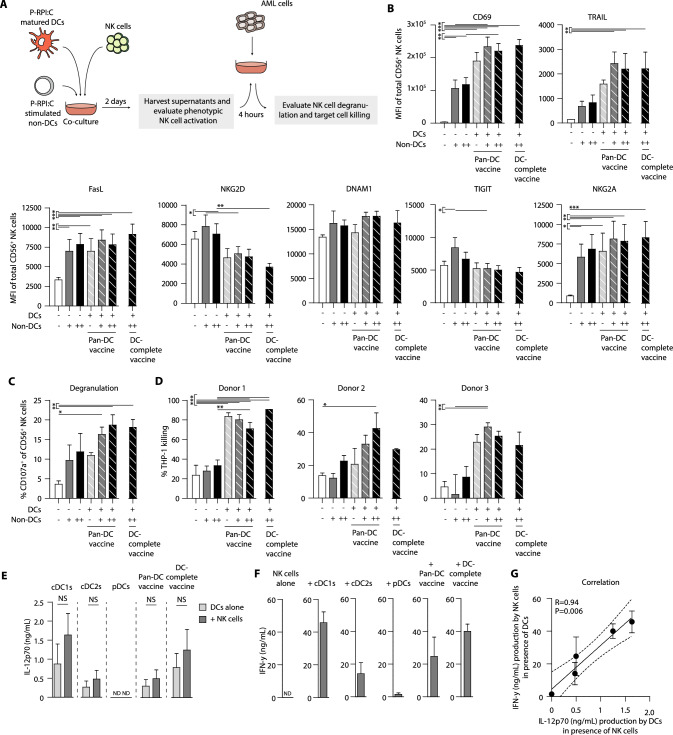
DC-mediated NK cell activation and cytolytic capacity is not hampered by the presence of non-DC. **a** Schematic overview of experimental setup. Mature DCs were co-cultured with healthy donor NK cells (DC-NK cell ratio 1:1) in the absence or presence of P-RPI:C stimulated non-DCs (DC:non-DC ratio 1:1 or 1:2) for 2 days. Subsequently, THP-1 cells were added for 4 h. NK cell assays were performed for multiple independent donors (*n* = 4 in b; *n* = 3 in c-g). **b** MFI of activating and inhibitory molecules on NK cells after 2 days of DC-NK cell co-culture *(mean* ± *SEM)*. **c** Degranulation of DC-activated NK cells in the absence or presence of TLR-stimulated non-DCs upon 4 h exposure to THP-1 cells *(mean* ± *SEM)*. **d** Killing of CFSE-labeled THP-1 cells by DC-activated NK cells in the absence or presence of TLR-stimulated non-DCs *(mean* ± *SD)*. **e–f** Release of pro-inflammatory cytokine IL-12p70 **e** and IFN- γ **f** by NK cells after 2 day co-culture with the different DC vaccines *(mean* ± *SEM)*. **g** Correlation of IL-12p70 and IFN-γ production during DC-NK cell co-cultures *(mean* ± *SEM)*. Statistical analyses was performed using a repeated measures one-way ANOVA followed by Bonferroni correction comparing selected pairs of means **b–c**, an one-way ANOVA followed by Bonferroni correction comparing selected pairs of means **d**, paired T-tests **e** and a Pearson correlation **g**. *P < 0.05, **P < 0.01, ***P < *0.001.* ND, not detectable. *DC* dendritic cell, *NK cell* natural killer cells, *cDC* conventional dendritic cell, *pDC* plasmacytoid dendritic cell; non-DC, non-end-stage dendritic cell, *P-RPI:C* CpG-P + R848 + Poly I:C, *CFSE* carboxyfluorescein succinimidyl ester, *SEM* standard error of the mean, *MFI* median fluorescence intensity

In addition to T cells, NK cells are the other key immune effector population responsible for the therapeutic GVT effect in alloSCT patients [31, 32]. We previously demonstrated the profound NK cell stimulatory capacity of our CD34^+^ HPC-derived DC subsets, leading to NK activation and subsequent killing of AML cells [24]. Here, we investigated the impact of non-DC presence on DC-mediated NK cell activation (Fig. 6a).

Our findings revealed that NK cells co-cultured with non-DCs exhibited increased expression of the activation markers CD69, TRAIL, FasL, and NKG2D, as well as NK cell inhibitory receptors TIGIT and NKG2A (Figs. 6b, S7a–c). Importantly, the addition of cDCs/pDCs further augmented expression levels of CD69 and TRAIL, while maintaining TIGIT and DNAM-1 expression at baseline, and decreasing NKG2D expression. Although non- DC-activated NK cells effectively degranulated upon exposure to AML cell line THP-1, this alone was insufficient.

Previously, it has been demonstrated that a cDC1 and NK cell gene signature in the tumor microenvironment correlates positively with patient survival [33]. Amongst others, this might be attributed to the bi-directional cross-talk between cDC1s and NK cells, mainly facilitated by DC-derived IL-12 and NK cell-derived IFN-γ [34]. Therefore, we interrogated the cross-talk between our DCs and NK cells in more detail by examining cytokine production upon DC subset and NK cell co-culture. We observed a trend towards increased production of IL-12p70 when mature cDCs were cultured in the presence of NK cells (Fig. 6e), while IFN-α production of pDCs remained unchanged (Fig. S7d). Conversely, cDCs effectively induced NK cell activation, as demonstrated by high production of IFN-γ (Fig. 6f). This effect was less pronounced in pDC-NK cell co-cultures. Intriguingly, a strong correlation between cDC-derived IL-12p70 and NK cell-derived IFN-γ was demonstrated (Fig. 6g), while no significant correlation was found between pDC-derived IFN-α and NK cell-derived IFN-γ (Fig. S7e).

To summarize, although non-DCs can activate NK cells to some extent, the productive NK cell activation and subsequent tumor reactivity primarily depend on the presence of DC subsets. These results highlight the potency of the DC-complete vaccine in effectively inducing anti-tumor NK cell responses in vitro.

### DC-complete vaccination effectively activates NK cells in vivo

Next, we investigated the in vivo NK cell activating potential of our DC-complete vaccine. IL-15 co-administration i.p. was essential to support NK cell engraftment in blood, spleen, bone marrow and liver (S8a–c). This also led to modest NK cell activation, as evidenced by increased expression of CD56, NKG2A and NKp46. More importantly, DC-complete vaccination induced superior expression of NK cell activation marker CD69 at day 2, which returned to baseline over time (Fig. S8d–g).

**Fig. 7:**
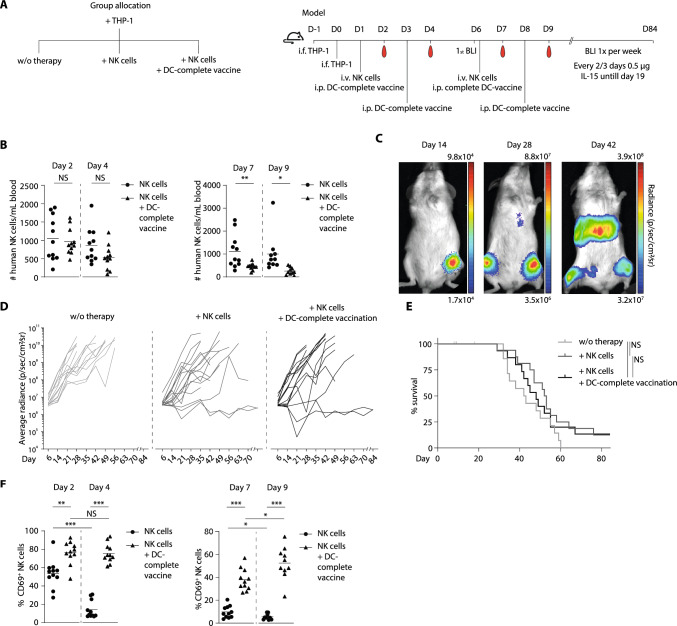
In vivo DC-complete vaccination strongly boosted and effectively retained phenotypic NK cell activation. **a** Schematic overview of group allocation and experimental setup of in vivo NK cell model. Mice were i.f. injected with 2 × 10^4^ LucGFP THP-1 cells, followed by two i.v. NK cell infusions (3 × 10^6^) or PBS as control and four i.p. treatments with DC-complete vaccine (0.5 × 10^6^ end-stage-DCs) or PBS as control (*n* = 14–16 mice per group, with *n* = 11 mice per group per time-point for phenotype analysis (**b**, **f**) due to welfare restrictions in blood volume withdrawal). **b** Absolute numbers of human NK cells per mL blood at day 2 and 4 (after 1^st^ NK cell injection) or day 7 and 9 (after 2^nd^ NK cell injection). **c** Example of metastasis formation after i.f. tumor injection in the left hind leg.** d** BLI results from each individual mouse and **e** survival of all three treatment groups. **f** Percentage of CD69 positive NK cells at day 2 and 4 (after 1^st^ NK cell injection) and day 7 and 9 (after 2^nd^ NK cell injection). Statistical analyses was performed using non-parametric unpaired T-tests **b–f** and Log-rank (Mantel-Cox) test **e**. *P < 0.05, **P < 0.01,***P < *0.001*. *NK cell* natural killer cell, *BLI* bioluminescence imaging, *i.f.* intrafemoral, *Luc* luciferase, *GFP* green fluorescent protein

To further examine the in vivo NK cell stimulatory capacity of our DC-complete vaccine, we investigated whether multiple vaccinations could maintain the active NK cell phenotype using a LucGFP THP-1 tumor xenograft model [35]. Mice were i.f. injected with the AML cell line LucGFP THP-1, followed by two i.v. NK cell infusions and four i.p. DC-complete vaccinations (Fig. 7a). Both NK cells and the DC-complete vaccine demonstrated functionality prior to infusion, as validated in in vitro assays (Fig. S9a–d). The absolute number of NK cells in blood was similar at days 2 and 4, but was lower at days 7 and 9 in the DC-complete vaccinated mice compared to NK cell only treated mice, which may indicate increased extravasation in DC-vaccinated mice (Fig. 7b). Despite this, increased tumor control was not yet consistently observed in the DC-complete vaccinated group. Tumor control was noted in some mice with low initial tumor burden, as reflected by early tumor disappearance or late tumor onset in the NK cell treated groups (Fig. 7c–e, S9i–k). Most importantly, even in the presence of THP-1 cells, we again observed strongly enhanced NK cell activation following each DC-complete vaccination (Figs. 7f, S9e–h), as reflected by increased expression of activation marker CD69. In contrast, non-vaccinated mice treated with NK cells only lost their initial NK cell activation signature (CD69^+^) between day 2 to day 4, while this activation was retained in mice treated with a second DC-complete vaccination (Fig. 7f). To conclude, DC-complete vaccination effectively induced in vivo NK cell activation.

## Discussion

Despite the power of GVT immune responses and the curative potential of alloSCT in high-risk hematological malignancies, relapse remains the major cause of treatment failure [1, 2]. This might be attributed to inadequate tumor reduction prior to alloSCT or insufficient induction and/or functionality of GVT immune responses [3]. Therefore, there is a medical need for adjuvant therapies to improve outcome. DC vaccination is a highly attractive strategy to boost innate and adaptive GVT immunity. In the last decades, most vaccination strategies in cancer patients, including hematological malignancies, focused on moDCs [5]. Despite induction of tumor-reactive immunity, the clinical efficacy remains modest, which might be attributed to their suboptimal migratory capacity, survival and/or T cell priming potential [4]. Alternatively, naturally occurring blood DC subsets, including cDC1s, cDC2s and pDCs, are postulated to be more powerful due to their unique functional properties and cross-talk capacity [4, 6, 36]. By potentiating each other, robust induction of innate and adaptive immune responses can be achieved [17–23, 37–39]. Together, this emphasizes the clinical attractiveness of a pan-DC vaccine (i.e. combined administration of cDC1s, cDC2s and pDCs). To produce high numbers of DCs, we have previously developed a unique clinically applicable culture process which allows eventual large scale generation of highly functional end-stage-blood DC subsets (cDC1s, cDC2s and pDCs) from a small fraction (< 5%) of the donor stem cell graft. From only 10 × 10^6^ G-CSF mobilized HPCs we can generate up to 220 × 10^6^ cDC2s, 100 × 10^6^ pDCs and 63 × 10^6^ cDC1s [24]. Besides blood DC subsets, our DC-complete vaccine still contains other cells, so called non-end-stage DCs (i.e. non-DCs). To allow eventual clinical translation of this DC-complete vaccine, we therefore delineated the cellular composition and investigated the potential immunomodulatory effects of non-DCs on DC-mediated T and NK cell activation in in vitro and in vivo models.

G-CSF-mobilized CD34^+^ progenitor cells are the most commonly used stem cell source for transplantation of adult patients with hematological malignancies [40]. G-CSF signaling skews mobilized stem and progenitor cell differentiation towards myelopoiesis [41, 42]. Accordingly, we observed low CLP, and high CMP and GMDP frequencies at the start of our culture. During the first week of the culture, we observed CMP to GMDP differentiation, in which FLT3L, present during the whole culture, plays an important role [43]. Subsequently, during the second week, MEPs disappeared due to omittance of TPO, an essential factor for megakaryocytic development [44, 45]. A marked increase in monocytic/dendritic lineage committed MDPs was observed, which may be attributed to GM-CSF and FLT3L-mediated signaling (through CD116 and CD135, respectively) and IFN-α-induced CD135 expression [43, 46, 47]. Interestingly, CDPs appear from day 5 onwards with a relatively stable prevalence over time, preceding the detection of cDCs and pDCs. At the end of our culture process, approximately 20% of the DC-complete vaccine consists of CD34^+^ cells. MDPs are the most prevalent, suggesting ongoing end-stage-DC development, which may facilitate continuous in vivo development upon infusion with the DC-complete vaccine.

Besides the differentiation of CD34^+^ progenitor cells into more committed progenitor cells and eventual end-stage-DC subsets, also other cell populations arose, which accounted for approximately 47% of our DC-complete vaccine. We demonstrated that the CD34^−^ non-DC compartment is heterogeneous and comprised of pre-cDCs, granulocyte- and monocyte-like cells at various developmental stages. Interestingly, upon P-RPI:C stimulation, a large proportion of the non-DC compartment profoundly induced expression of co-stimulatory molecules and produced significant levels of the pro-inflammatory cytokine IL-12p70, which is an indication of APC-features by (part of) those cells [48, 49]. This may be attributed to the presence of pre-cDCs and monocyte-like cells in our non-DC fraction. In follow-up studies, intracellular cytokine staining may further pinpoint which fraction of the non-DC compartment is responsible for the IL-12p70 production after TLR-stimulation in co-culture experiments. Accordingly, we demonstrated that non-DCs can elicit antigen-specific CD8^+^ T cell responses by themselves and further augmented the T cell stimulatory capacity of end-stage-DC subsets in an antigen-independent manner. This may, amongst others, be mediated by the non-DC-derived IL-12p70, which plays a key role in T cell priming and activation [50–53]. Although non-DCs alone induced some antigen-specific T cell expansion, mature end-stage DC subsets were superior. We demonstrated that all end-stage DC subsets can be effectively matured using one TLR-cocktail containing CpG-P, R848 and Poly I:C (together P-RPI:C). CpG-P did not negatively affect cDC maturation nor IL-12p70 production. Although, P-RPI:C stimulation resulted in a reduction in IFN-α production by pDCs, this did not impair pDC-mediated T and NK cell activation. Most importantly, our P-RPI:C matured and peptide-loaded DC-complete vaccine strongly induced expansion of antigen-specific memory CD8^+^ T cells both in vitro and in vivo. Interestingly, upon DC-complete vaccination in vivo these T cells exhibited a dominant effector memory phenotype (CCR7^−^CD45RA^−^) in contrast to a more terminally differentiated effector memory phenotype (CCR7^−^CD45RA^+^) in pan-DC vaccine treated mice. As T_em_ cells have superior proliferative capacity and tumoricidal effector function over T_emra_ cells, this may result in more powerful and long-lasting anti-tumor responses [54]. Besides T cells, NK cells play an important role in the GVT effect [31, 32]. Here, we demonstrated reciprocal DC-NK cell interactions. These findings are in line with previous publications demonstrating cDC-NK cell cross-talk [55, 56]. The interaction of DCs and NK cells through CX3CR1-CX3CL1 has been described to augment IFN-γ production by NK cells [57]. Accordingly, high CX3CR1 expression by part of our cDC1s and cDC2s may have contributed to enhanced NK-mediated IFN-y secretion. Notably, similar to the T cell assays, we observed that non-DCs alone were capable of inducing NK cell activation. Again, superior effects were found when non-DCs were combined with end-stage-DC subsets, resulting in productive NK cell activation and tumor killing capacity in vitro. Unfortunately, the DC-mediated NK cell activation in vivo did not yet result in a superior anti-tumor response in the aggressive THP-1 tumor model [58]. Future research will focus on further optimization of this in vivo model by using a less aggressive tumor either with a higher sensitivity to NK cell-mediated killing, such as K562 or HL60, or by improving the treatment window through a lower tumor load [24]. Most importantly, in vivo DC-complete vaccination induced profound phenotypic activation of NK cells, which was further augmented upon multiple vaccinations.

In conclusion, we demonstrated that the presence of non-DCs did not negatively impact pDC and cDC potency, and even augmented DC-mediated antigen-specific CD8^+^ T cell expansion. Although the DC-complete vaccine is heterogeneous and varies in composition between CD34^+^ HPC donors, all individual products exerted robust functional potency both in in vitro and in vivo models. Our DC-complete vaccine exhibited high expression of co-stimulatory molecules and effectively produced pro-inflammatory cytokines IL-12p70 and IFN-α upon combined TLR3/7/8 stimulation. More importantly, DC-complete vaccination effectively boosted antigen-specific CD8^+^ T cell expansion, NK cell activation and NK tumor reactivity in vitro*.* Accordingly, in vivo DC-complete vaccination potently induced expansion of antigen-specific CD8^+^ T_em_ cells and strongly activated NK cells. Together, these data demonstrate the feasibility and potency of our DC-complete vaccine to boost tumor-reactive immune responses. Hereby, additional time-consuming and expensive DC-isolation procedures can be avoided. This provides rationale for further clinical translation of our DC-complete vaccine to augment innate and adaptive GVT immunity in order to induce long-lasting tumor control and improve survival of hemato-oncology patients post alloSCT.

### Supplementary Information

Below is the link to the electronic supplementary material.

Supplementary file1 (DOCX 28 KB)

Supplementary file2 (PDF 5706 KB)

Supplementary file3 (PDF 27781 KB)

Supplementary file4 (PDF 20409 KB)

Supplementary file5 (PDF 497 KB)

Supplementary file6 (PDF 642 KB)

Supplementary file7 (PDF 667 KB)

Supplementary file8 (PDF 662 KB)

Supplementary file9 (PDF 935 KB)

Supplementary file10 (PDF 2780 KB)

## Data Availability

The datasets used and analyzed during the current study are available from the corresponding author on reasonable request.
