# Supplementary material for: Human CD34+-derived complete plasmacytoid and conventional dendritic cell vaccine effectively induces antigen-specific CD8+ T cell and NK cell responses in vitro and in vivo
Source: Cell Mol Life Sci. 2023 Sep 20;80(10):298. doi: 10.1007/s00018-023-04923-4 (PMC10511603; doi:10.1007/s00018-023-04923-4)
Supplement: Supplementary file 1 — Supplementary file1 (DOCX 28 KB) [file 18_2023_4923_MOESM1_ESM.docx]

***Supplementary Table 1. Patient characteristics.***

| **Patient** | **Disease** | **Time point sample** | **Sample type** | **MiHA** | **% MiHA-specific CD8^+^ T cells at day 0** |
| --- | --- | --- | --- | --- | --- |
| **A** | AML | 12 months post alloSCT | PB | HA-1 | 0.02% |
| **B** | MDS | 9 months post alloSCT | PB | HA-1 | 0.25% |

*AML, acute myeloid leukemia; MDS, myeloid dysplastic syndrome; alloSCT, allogeneic stem cell transplantation; PB, peripheral blood; MiHA, minor histocompatibility antigen*

***Supplementary Table 2. Overview of FCM panels and antibodies used***

| **Specificity** | **Fluorochrome** | **Clone** | **Isotype** | **Company** | **Catalogue #** |
| --- | --- | --- | --- | --- | --- |
| ***Stem cell panel*** | | | | | |
| CD34 | BV785 | 561 | Mouse IgG2a | BioLegend | 343626 |
| CD90 | PE-Cy5 | E10 | Mouse IgG1 | BioLegend | 328112 |
| CD123 | BV510 | 4G8 | Mouse IgG1 | BD Biosciences | 743338 |
| CD115 | BV711 | 9-4D2-1E4 | Rat IgG1 | BioLegend | 347318 |
| CD117 | BV605 | 104D2 | Mouse IgG1 | BioLegend | 313218 |
| CD123 | PE | 6H6 | Mouse IgG1 | BioLegend | 306006 |
| CD38 | PE-Dazzle594 | HIT2 | Mouse IgG1 | BioLegend | 303538 |
| CD10 | BV421 | HI10a | Mouse IgG1 | BioLegend | 312218 |
| CD45 | APC-Fire750 | 2D1 | Mouse IgG1 | BioLegend | 268517 |
| CD105 | BB700 | 266 | Mouse IgG1 | BD Biosciences | 566528 |
| CD116 | FITC | 4H1 | Mouse IgG1 | BioLegend | 305906 |
| HLA-DR | BUV737 | G46-6 | Mouse IgG2a | BD Biosciences | 748339 |
| CD45RA | AF647 | HI100 | Mouse IgG2b | BioLegend | 304154 |
| Live/dead | ViaKrome808 | - | - | Beckman Coulter | C36628 |
| ***Dendritic cell panel*** | | | | | |
| CD34 | BV785 | 561 | Mouse IgG2a | BioLegend | 343626 |
| XCR1 | FITC | S1506E | Rat IgG2a | BioLegend | 372612 |
| CD1c | APC-Cy7 | L161 | Mouse IgG1 | BioLegend | 331520 |
| HLA-DR | BV510 | L243 | Mouse IgG2a | BioLegend | 307646 |
| CD2 | BUV737 | RPA-210 | Mouse IgG1 | BD Biosciences | 741821 |
| CD11c | BUV395 | B-Ly6 | Mouse IgG1 | BD Biosciences | 563787 |
| CD14 | PerCP-Cy5.5 | M5E2 | Mouse IgG2a | BioLegend | 301824 |
| CD123 | PE-Cy7 | 6H6 | Mouse IgG1 | BioLegend | 306010 |
| CD141 | BV605 | M80 | Mouse IgG1 | BioLegend | 344118 |
| CD303 | BV711 | 201A | Mouse IgG2a | BioLegend | 354234 |
| CX3CR1 | PE-Dazzle594 | 2A9-1 | Rat IgG2b | BioLegend | 341624 |
| CD370 | PE | 8F9 | Mouse IgG2a | BioLegend | 353804 |
| CD19 | PE-Cy5 | J3-119 | Mouse IgG1 | Beckman Coulter | A07771 |
| CD45RA | AF647 | HI100 | Mouse IgG2b | BioLegend | 304154 |
| CD33 | BV421 | WM53 | Mouse IgG1 | BioLegend | 030416 |
| Live/dead | ViaKrome808 | - | - | Beckman Coulter | C36628 |
| ***Mono/Myeloid panel*** | | | | | |
| CD34 | BV785 | 561 | Mouse IgG2a | BioLegend | 343626 |
| CD197 | PE-Cy78 | G043H7 | Mouse IgG2a | BioLegend | 353226 |
| CD13 | BUV737 | WM15 | Mouse IgG1 | BD Biosciences | 741828 |
| CD117 | BV605 | 104D2 | Mouse IgG1 | BioLegend | 313218 |
| CD163 | PE-CF594 | GHI/61 | Mouse IgG1 | BD Biosciences | 562670 |
| CD206 | BV711 | 15-2 | Mouse IgG1 | BioLegend | 321136 |
| CD36 | PerCP-Cy5.5 | 5-271 | Mouse IgG2a | BioLegend | 336224 |
| CD38 | PE-Cy5 | HIT2 | Mouse IgG1 | BioLegend | 303507 |
| CD16 | BUV496 | 3G8 | Mouse IgG1 | BD Biosciences | 612944 |
| CD15 | APC-Fire750 | W6D3 | Mouse IgG1 | BioLegend | 323042 |
| CD19 | BV510 | HIB19 | Mouse IgG1 | BioLegend | 302242 |
| CD14 | FITC | HCD14 | Mouse IgG1 | BioLegend | 325604 |
| CD45 | BUV395 | HI30 | Mouse IgG1 | BD Biosciences | 563792 |
| HLA-DR | APC-R700 | G46-6 | Mouse IgG2a | BD Biosciences | 565127 |
| CD3 | BV510 | OKT3 | Mouse IgG2a | Beckman Coulter | 317332 |
| CD33 | BV421 | WM53 | Mouse IgG1 | BioLegend | 303416 |
| CD371 | APC | 50C1 | Mouse IgG2a | BioLegend | 353606 |
| CD11b | PE | Bear1 | Mouse IgG1 | Beckman Coulter | IM2581U |
| Live/dead | ViaKrome808 | - | - | Beckman Coulter | C36628 |
| ***Non-DC panel*** | | | | | |
| CD303 | BUV496 | V24-785 | Mouse IgG1 | BD Biosciences | 749921 |
| CD34 | BV510 | 581 | Mouse IgG1 | BioLegend | 343527 |
| CD13 | BUV737 | WM15 | Mouse IgG1 | BD Biosciences | 741828 |
| CD163 | PE-CF94 | GHI/61 | Mouse IgG1 | BD Biosciences | 562670 |
| CD123 | PE-Cy5 | 6G6 | Mouse IgG1 | BioLegend | 306008 |
| CD141 | PE-Cy7 | 0 | Mouse IgG1 | BioLegend | 344109 |
| CD206 | BV711 | 15-2 | Mouse IgG1 | BioLegend | 321136 |
| CD36 | PerCP-Cy5.5 | 5-271 | Mouse IgG2a | BioLegend | 336224 |
| CD15 | APC-Fire750 | W6D3 | Mouse IgG1 | BioLegend | 323042 |
| CD11c | BUV395 | B-Ly6 | Mouse IgG1 | BD Biosciences | 563787 |
| CD370 | PE | 8F9 | Mouse IgG2a | BioLegend | 353804 |
| CD19 | BV510 | HIV19 | Mouse IgG1 | BioLegend | 302242 |
| CD14 | FIC | HCD14 | Mouse IgG1 | BioLegend | 325604 |
| HLA-DR | APC-R700 | G46-6 | Mouse IgG2a | BD Biosciences | 565127 |
| CD45RA | AF647 | HI100 | Mouse IgG2b | BioLegend | 304154 |
| CD11b | BV785 | ICRF44 | Mouse IgG1 | BioLegend | 301345 |
| CD1c | BV605 | L161 | Mouse IgG1 | BioLegend | 331537 |
| CD3 | BV510 | OKT3 | Mouse IgG2a | BioLegend | 317332 |
| CD33 | BV421 | WM53 | Mouse IgG1 | BioLegend | 303416 |
| Live/dead | ViaKrome808 | - | - | Beckman Coulter | C36628 |
| **Dendritic cell maturation** | | | | | |
| CD1c | APC-Cy7 | L161 | Mouse IgG1k | BioLegend | 331520 |
| CD123 | BV510 | 6H6 | Mouse IgG1k | BioLegend | 306022 |
| CD141 | APC | M80 | Mouse IgG1k | BioLegend | 344106 |
| CD80 | PE-Cy7 | L307.4 | Mouse IgG1k | BD Biosciences | 561135 |
| CD83 | PE | 2D10 | Mouse IgG1k | BioLegend | 305208 |
| CD86 | AF488 | IT2.2 | Mouse IgG2b | BioLegend | 305414 |
| Live/dead | Sytox Blue | - | - | Thermo Fisher | S34859 |
| ***In vitro T cell assays*** | | | | | |
| CD3 | PE-C7 | UCHT1 | Mouse IgG1k | BioLegend | 300420 |
| CD8 | FITC | SK1 | Mouse IgG | BioLegend | 344704 |
| Tetramer | PE | - | - | LUMC, M. Kester | customized |
| Tetramer | APC | - | - | LUMC, M. Kester | customized |
| Live/dead | Sytox blue | - | - | Thermo Fisher | S34859 |
| ***In vitro NK cell assays*** | | | | | |
| CD69 | FITC | FN50 | Mouse IgGk | BioLegend | 310904 |
| CD253/TRAIL | APC | RIK-2 | Mouse IgG1k | BioLegend | 308210 |
| CD178/FasL | PE | NOK-1 | Mouse IgG1k | BioLegend | 306407 |
| CD314/NKG2D | BV785 | 1D11 | Mouse IgG1k | BioLegend | 320832 |
| CD159a/NKG2A | PE-Cy7 | Z199 | IgG2bk | Beckman Coulter | PN B10246 |
| DNAM1 | BV510 | 11A8 | IgG1k | BioLegend | 338329 |
| TIGIT | BV421 | A15153G | IgG2a | BioLegend | 372710 |
| CD56 | BV711 | HCD56 | Mouse IgG1k | BioLegend | 318335 |
| 7-AAD | - | - | - | Thermo Fisher | A1310 |
| *In vivo T cell experiment 1* | | | | | |
| CD3 | APC-Fire750 | UCHT1 | Mouse IgG1k | BioLegend | 300417 |
| CD8 | FITC | SK1 | Mouse IgG | BioLegend | 344704 |
| mCD45 | PECy7 | 30-F11 | Mouse IgG1k | BioLegend | 103114 |
| hCD45 | BV785 | HI30 | Mouse IgG1 | BioLegend | 304047 |
| CMV-tetramer | PE | - | - | LUMC, M. Kester | customized |
| CMV-tetramer | APC | - | - | LUMC, M. Kester | customized |
| Live-dead | ViaKrome808 | - | - | Beckman Coulter | C36638 |
| *In vivo T cell experiment 2* | | | | | |
| CD3 | APC-Fire750 | UCHT1 | Mouse IgG1k | BioLegend | 300417 |
| CD8 | FITC | SK1 | Mouse IgG | BioLegend | 344704 |
| mCD45 | PECy7 | 30-F11 | Mouse IgG1k | BioLegend | 103114 |
| hCD45 | BV785 | HI30 | Mouse IgG1 | BioLegend | 304047 |
| CD45RA | AF700 | HI100 | Mouse IgG2ak | BD Biosciences | 560673 |
| CD197/CCR7 | PE-CF594 | 150503 | Mouse IgG2ak | BioLegend | 562381 |
| CMV-tetramer | PE | - | - | LUMC, M. Kester | customized |
| CMV-tetramer | APC | - | - | LUMC, M. Kester | customized |
| Live/Dead | ViaKrome808 | - | - | Beckman Coulter | C36638 |
| *In vivo NK cell expansion assay* | | | | | |
| CD16 | BUV496 | 3G8 | Mouse IgG1k | BD Biosciences | 612944 |
| CD25 | BV421 | M-A251 | Mouse IgG1 | BD Biosciences | 562442 |
| hCD45 | BV785 | 30-F11 | Mouse IgG1k | BioLegend | 103114 |
| mCD45 | PECy7 | HI30 | Mouse IgG1 | BioLegend | 304047 |
| CD56 | BV711 | HCD56 | Mouse IgG1k | BioLegend | 318335 |
| CD69 | FITC | FN50 | Moue IgG1k | BioLegend | 310904 |
| NKG2A | APC | Z199 | Mouse IgG2b | Beckman Coulter | A60797 |
| TRAIL | PE | RIK-2 | Mouse IgG1k | BioLegend | 308206 |
| NKp46 | PE-Dazzle594 | 9E2 | Mouse IgG1k | BioLegend | 331930 |
| Live/Dead | ViaKrome808 | - | - | Beckman Coulter | C36638 |
| *In vivo NK cell tumor model* | | | | | |
| CD3 | APC-Fire750 | UCHT1 | Mouse IgG1k | BioLegend | 300417 |
| CD16 | BUV496 | 3G8 | Mouse IgG1k | BD Biosciences | 612944 |
| hCD45 | BV785 | 30-F11 | Mouse IgG1k | BioLegend | 103114 |
| mCD45 | PECy7 | HI30 | Mouse IgG1 | BioLegend | 304047 |
| CD56 | BV421 | HCD56 | Mouse IgG1k | BioLegend | 318327 |
| CD69 | FITC | FN50 | Moue IgG1k | BioLegend | 310904 |
| Live/Dead | ViaKrome808 | - | - | Beckman Coulter | C36638 |
