# Supplementary material for: Human CD34+-derived complete plasmacytoid and conventional dendritic cell vaccine effectively induces antigen-specific CD8+ T cell and NK cell responses in vitro and in vivo
Source: Cell Mol Life Sci. 2023 Sep 20;80(10):298. doi: 10.1007/s00018-023-04923-4 (PMC10511603; doi:10.1007/s00018-023-04923-4)
Supplement: Supplementary file 2 — Supplementary file2 (PDF 5706 KB) [file 18_2023_4923_MOESM2_ESM.pdf]

Supplementary figure 1

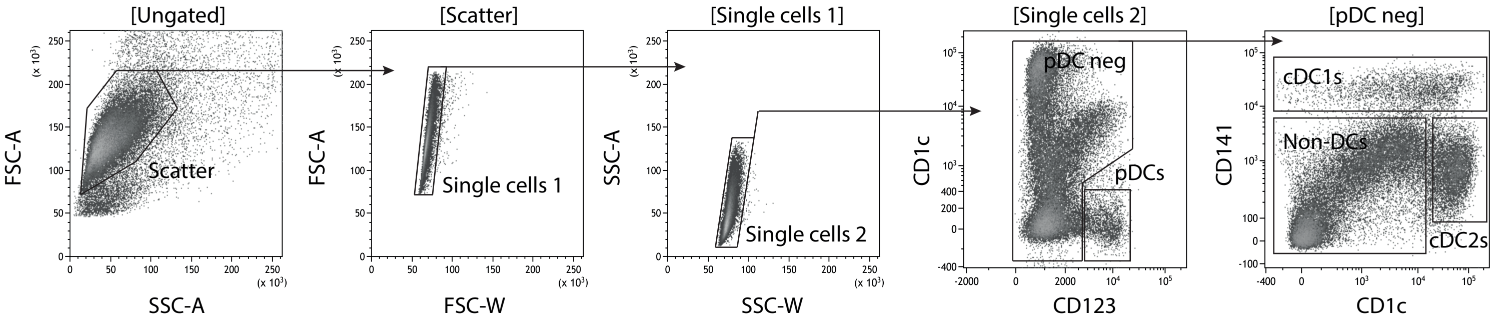

Supplementary figure 1. Representative flow cytometry plots visualizing sorting strategy of CD34⁺-derived pDCs, cDC1s, cDC2s and non-DCs. Cells are gated based on forward and side scatter characteristics, followed by doublet discrimination. pDCs are sorted based on CD123 expression and negativity for CD1c. After pDC exclusion, cDC1s are sorted based on high CD141 expression and cDC2s are sorted based on high CD1c expression. All remaining cells are sorted as non-DCs.
