# Supplementary material for: Human CD34+-derived complete plasmacytoid and conventional dendritic cell vaccine effectively induces antigen-specific CD8+ T cell and NK cell responses in vitro and in vivo
Source: Cell Mol Life Sci. 2023 Sep 20;80(10):298. doi: 10.1007/s00018-023-04923-4 (PMC10511603; doi:10.1007/s00018-023-04923-4)
Supplement: Supplementary file 3 — Supplementary file3 (PDF 27781 KB) [file 18_2023_4923_MOESM3_ESM.pdf]

Supplementary figure 2

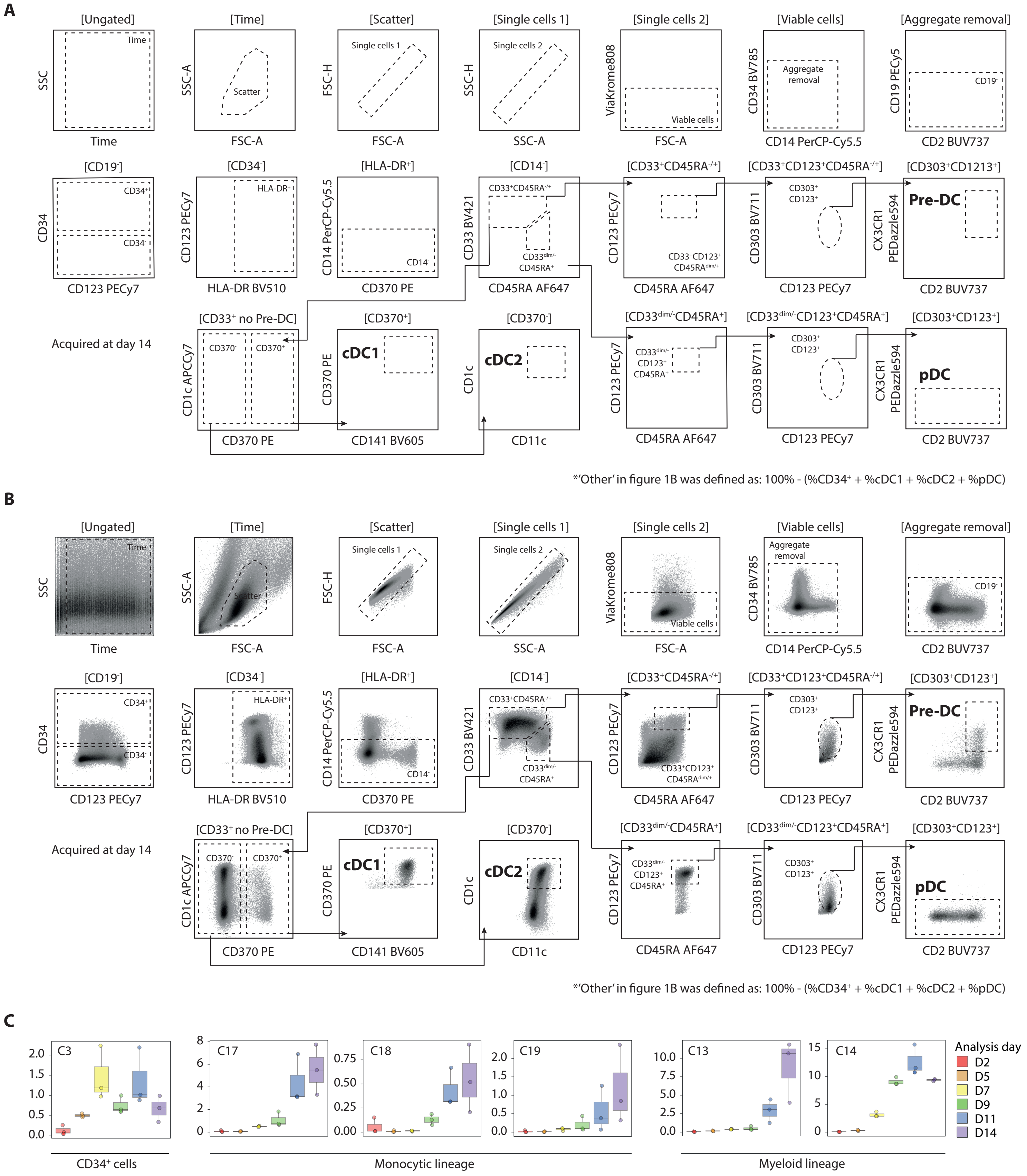

Supplementary figure 2. Schematic overview and representative flow cytometry plots visualizing the gating strategy for the identification of cDC1s, cDC2s, pDCs and pre-DCs. (a) Schematic representation of gating strategy. **(b)** Representative flow cytometry plots of gating strategy. **(c)** Frequency of metaclusters C3, C17, C19, C20, C13 and C14 (DC-panel) over time in box plots, organized by cell type based on respective marker expression visualized in fig1e (n=3).
