# Supplementary material for: Human CD34+-derived complete plasmacytoid and conventional dendritic cell vaccine effectively induces antigen-specific CD8+ T cell and NK cell responses in vitro and in vivo
Source: Cell Mol Life Sci. 2023 Sep 20;80(10):298. doi: 10.1007/s00018-023-04923-4 (PMC10511603; doi:10.1007/s00018-023-04923-4)
Supplement: Supplementary file 4 — Supplementary file4 (PDF 20409 KB) [file 18_2023_4923_MOESM4_ESM.pdf]

Supplementary figure 3

A

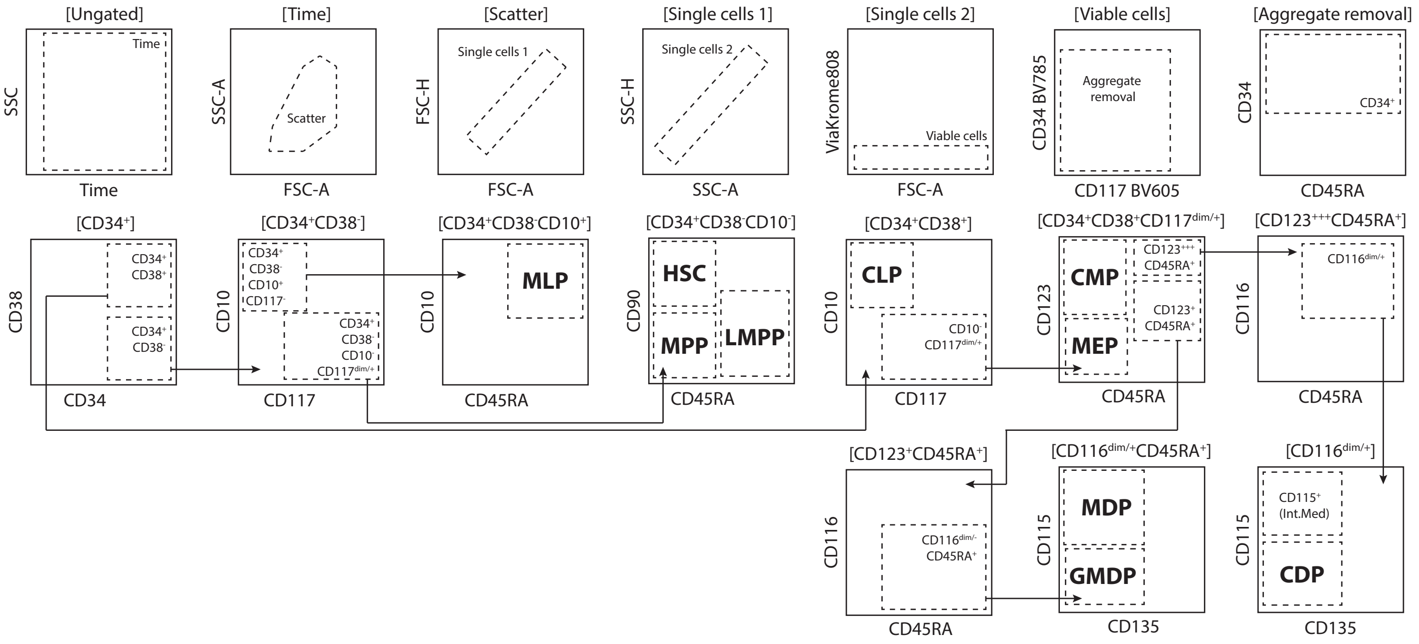

*Frequencies of progenitor subsets in figure 1C were defined within the CD34⁺ cells

B

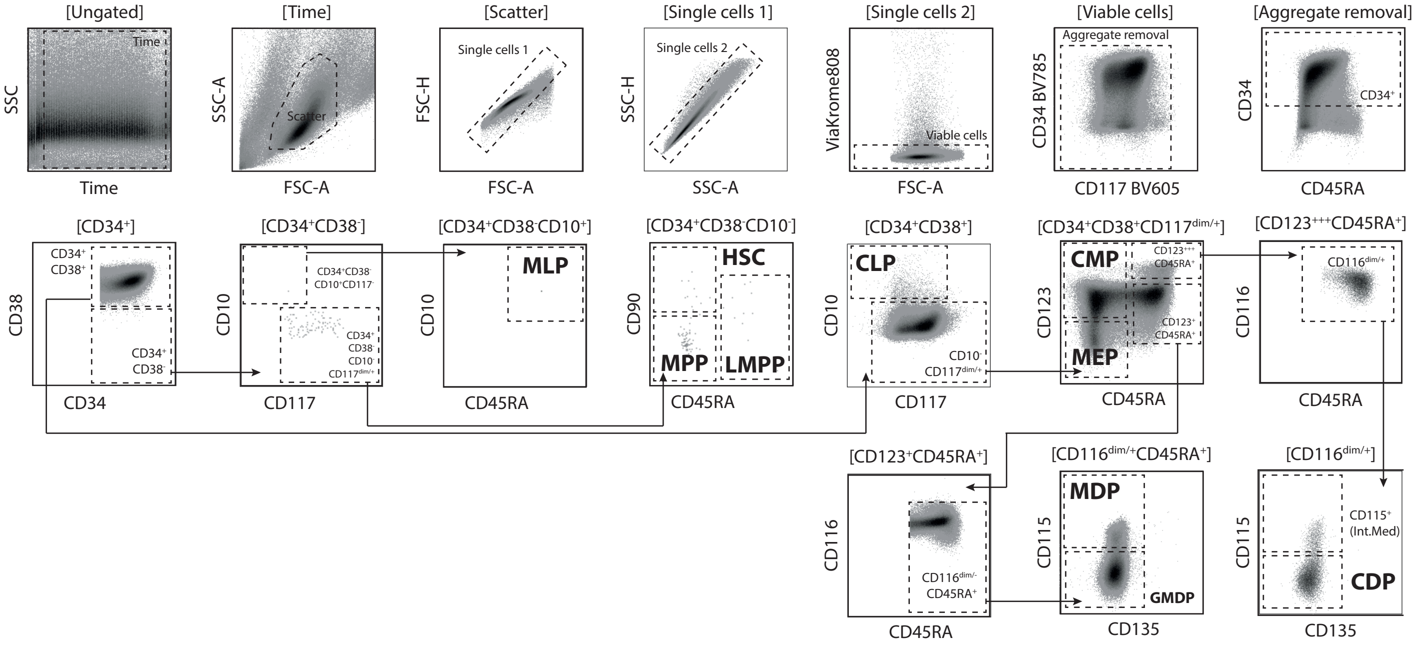

*Frequencies of progenitor subsets in figure 1C were defined within the CD34⁺ cells

Supplementary figure 3. Schematic overview and representative flow cytometry plots demonstrating the gating strategy for the identification of progenitor cell populations. (a) Schematic representation of gating strategy. **(b)** Representative flow cytometry plots of gating strategy.
