# Supplementary material for: Human CD34+-derived complete plasmacytoid and conventional dendritic cell vaccine effectively induces antigen-specific CD8+ T cell and NK cell responses in vitro and in vivo
Source: Cell Mol Life Sci. 2023 Sep 20;80(10):298. doi: 10.1007/s00018-023-04923-4 (PMC10511603; doi:10.1007/s00018-023-04923-4)
Supplement: Supplementary file 5 — Supplementary file5 (PDF 497 KB) [file 18_2023_4923_MOESM5_ESM.pdf]

Supplementary figure 4

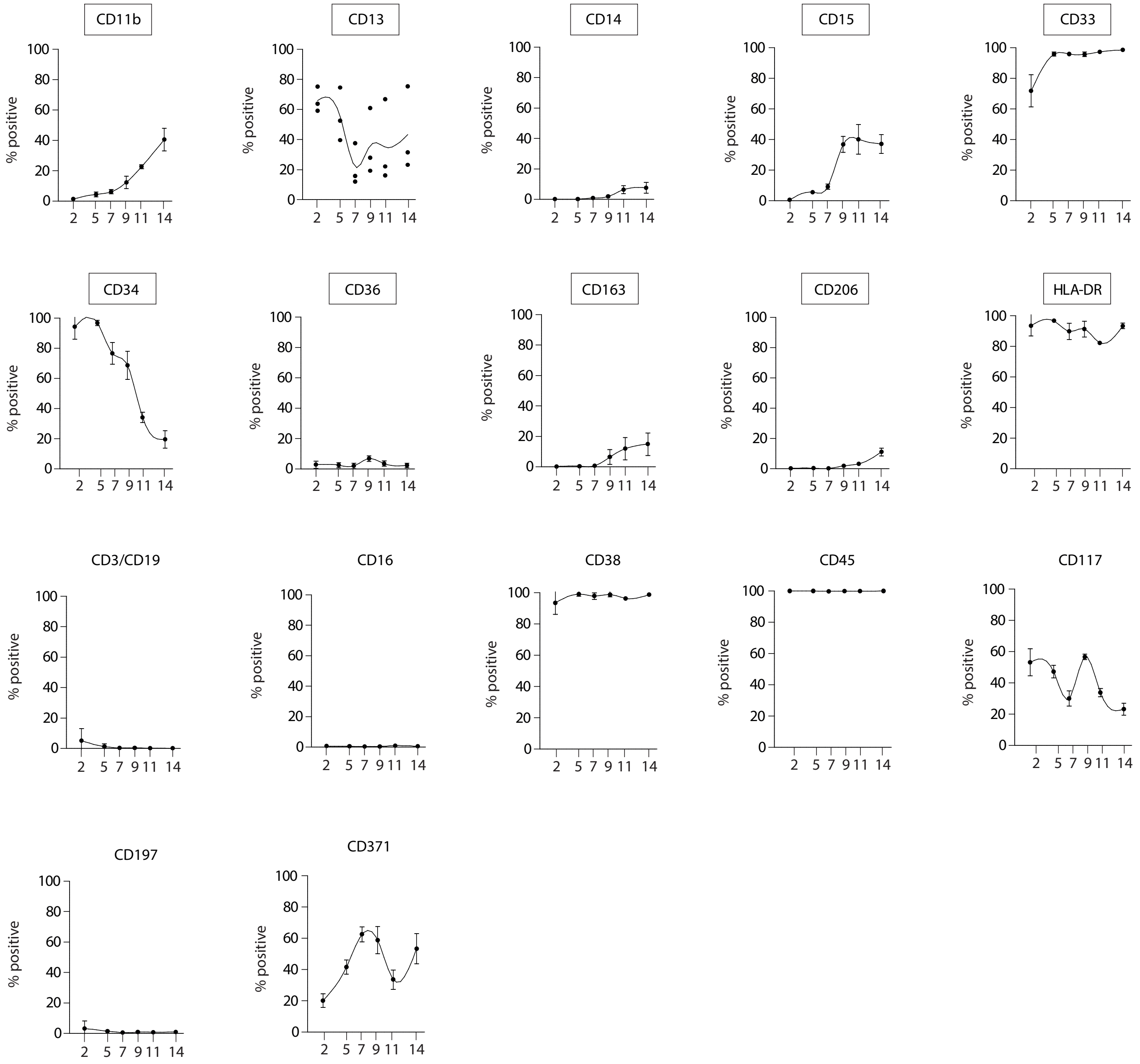

Supplementary figure 4. Frequencies of markers expressed on total viable cells in myeloid panel measured over time. Markers indicated in boxes were used to further characterize the non-DC compartment by using the non-DC panel.
