# Supplementary material for: Human CD34+-derived complete plasmacytoid and conventional dendritic cell vaccine effectively induces antigen-specific CD8+ T cell and NK cell responses in vitro and in vivo
Source: Cell Mol Life Sci. 2023 Sep 20;80(10):298. doi: 10.1007/s00018-023-04923-4 (PMC10511603; doi:10.1007/s00018-023-04923-4)
Supplement: Supplementary file 6 — Supplementary file6 (PDF 642 KB) [file 18_2023_4923_MOESM6_ESM.pdf]

Supplementary figure 5

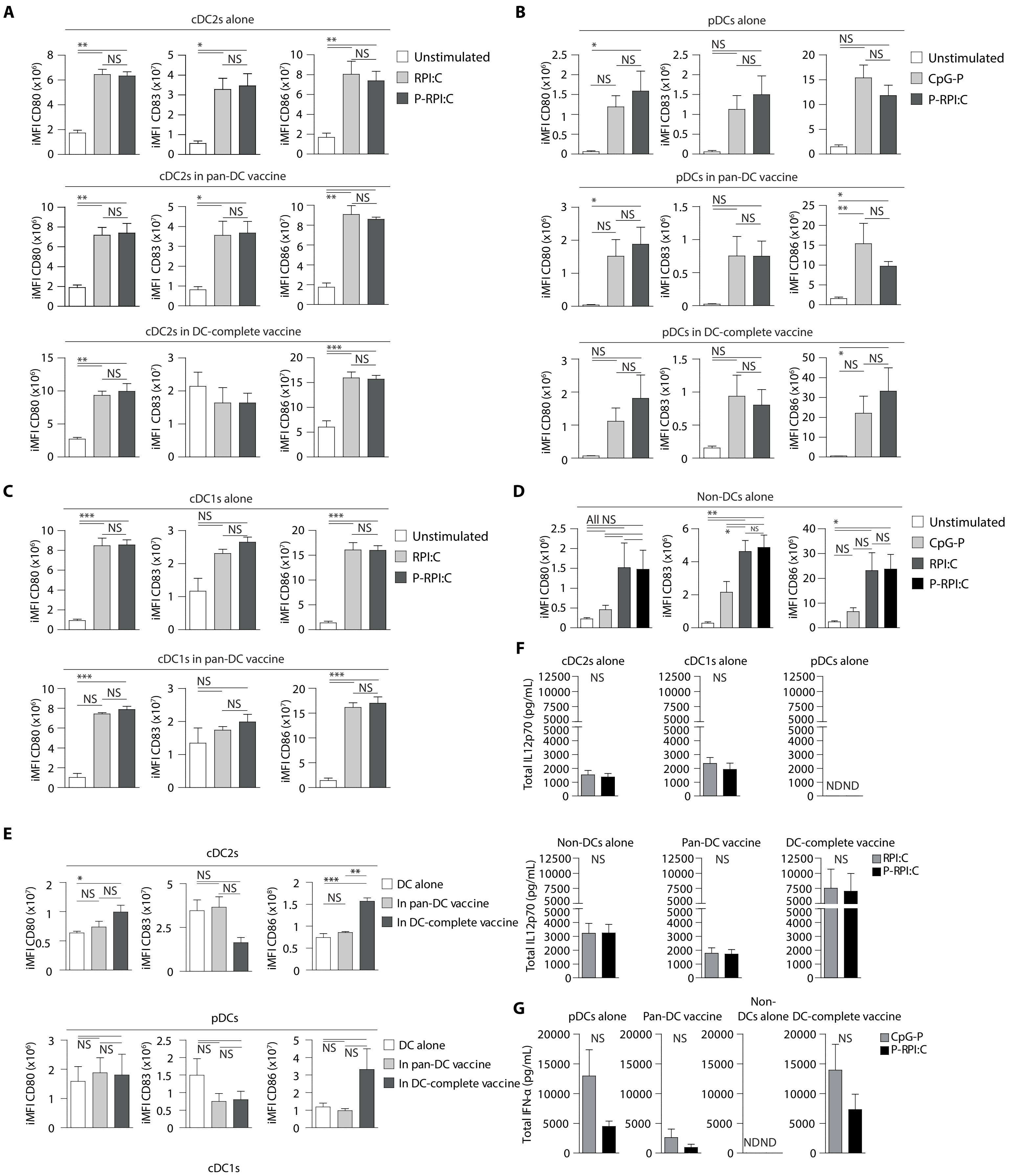

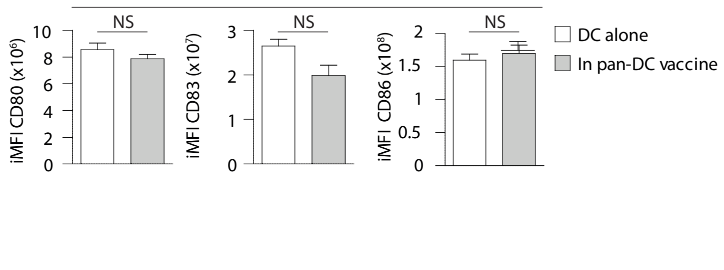

H

	cDC2s alone	cDC1s alone	pDCs alone	Non-DCs alone	Pan-DC vaccine	DC-complete vaccine
cDC2s	$25x10^3$	-	-	-	$7.3x10^3$	$7.3x10^3$
cDC1s	-	$25x10^3$	-	-	$3.1x10^3$	$3.1x10^3$
pDCs	-	-	$25x10^3$	-	$9.8x10^3$	$9.8x10^3$
Non-DCs	-	-	-	$96x10^3$	-	$96x10^3$
Total	$25x10^3$	$25x10^3$	$25x10^3$	$96x10^3$	$25x10^3$	$116x10^3$

Average # of cells and respective composition (n=3) of figure G and H

Supplementary Figure 5. non-DCs do not hamper TLR-stimulated cDC and pDC phenotypic and functional maturation. (a-d) iMFI of CD80, CD83 and CD86 on unstimulated or TLR-stimulated cells of the respective vaccination strategy. **(e)** iMFI of CD80, CD83 and CD86 on P-RPI:C stimulated cells of the respective vaccination strategy. **(f, g)** Release of pro-inflammatory cytokines IL-12p70 **(f)** and IFN-α **(g)** by TLR-stimulated cells of the respective vaccination strategy. **(h)** Table presenting the average absolute numbers of respective cDC2s, cDC1s, pDCs and non-DCs in each vaccination strategy used for figure f and g. Data is shown as mean ± SEM (n=3). Statistical analyses were performed using repeated measures one-way ANOVA followed by Bonferroni correction comparing all pairs of columns **(a-e)** or using a paired T-test **(f,g)**. **P<0.05, **P<0.01, ***P<0.001*.
