# Supplementary material for: Human CD34+-derived complete plasmacytoid and conventional dendritic cell vaccine effectively induces antigen-specific CD8+ T cell and NK cell responses in vitro and in vivo
Source: Cell Mol Life Sci. 2023 Sep 20;80(10):298. doi: 10.1007/s00018-023-04923-4 (PMC10511603; doi:10.1007/s00018-023-04923-4)
Supplement: Supplementary file 7 — Supplementary file7 (PDF 667 KB) [file 18_2023_4923_MOESM7_ESM.pdf]

Supplementary figure 6

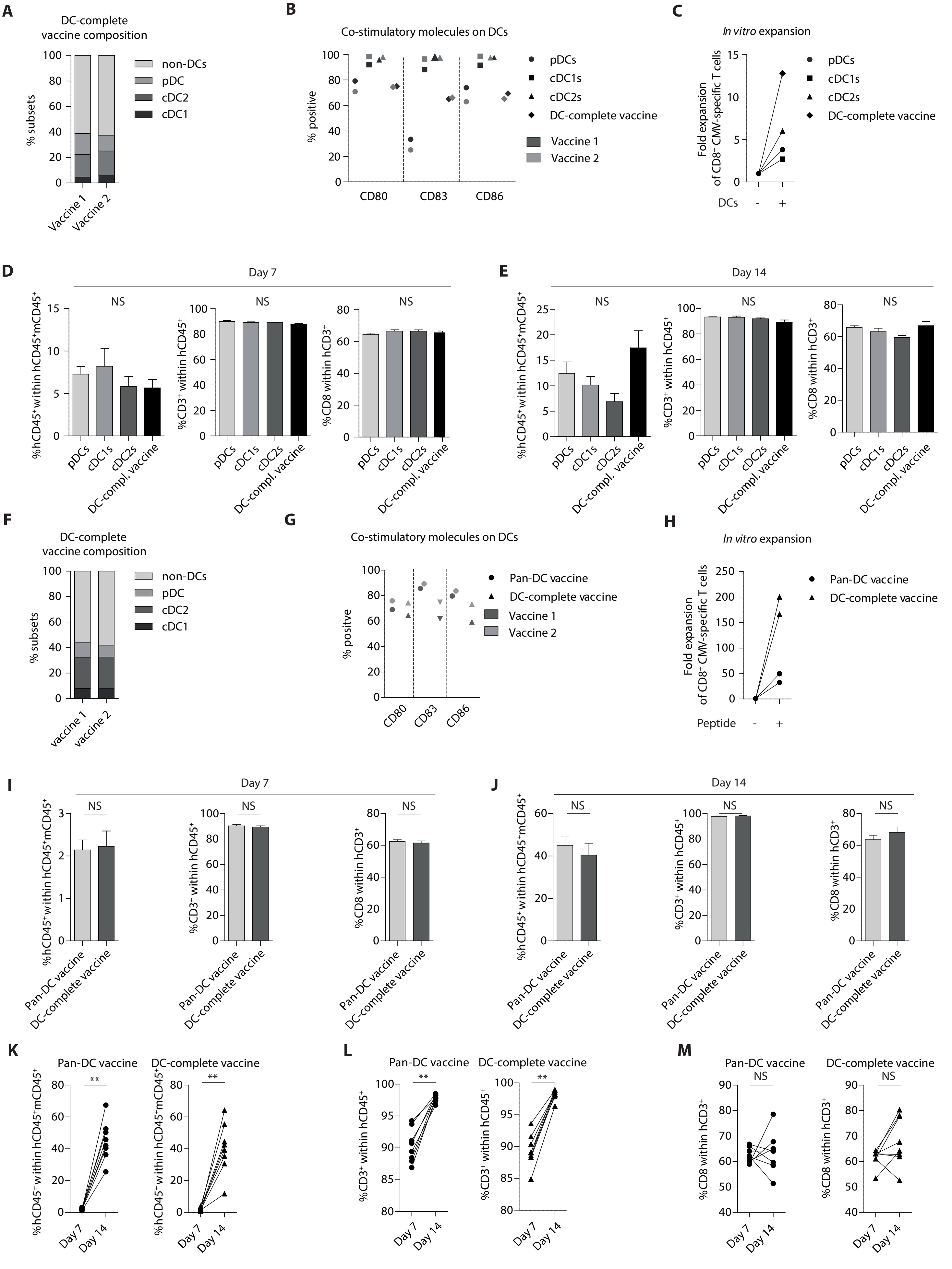

Supplementary figure 6. ***Ex vivo*** **evaluation of vaccine potency and engraftment of human immune cells in the** ***in vivo*** **T cell expansion model. (a)** Composition of DC-complete vaccine 1 and 2 used in *in vivo* T cell expansion model 1. **(b)** Expression of co-stimulatory molecules CD80, CD83 and CD86 on matured DC vaccine before *in vivo* injection. **(c)** *In vitro* evaluation of functional capacity of respective DC vaccines, used in *in vivo* experiments, to induce expansion of CMV-specific $CD8^+$ T cells. **(d, e)** Percentage of $hCD45^+$ cells, $CD3^+$ T cells ad $CD8^+$ T cells in vaccinated mice at day 7 **(d)** and day 14 **(e)**. **(f)** Composition of DC-complete vaccine 1 and 2 used in *in vivo* T cell expansion model 2. **(g)** Expression of co-stimulatory molecules CD80, CD83 and CD86 on matured DC vaccine before *in vivo* injection. **(h)** *In vitro* evaluation of functional capacity of pan-DC vaccine and DC-complete vaccine, used in *in vivo* experiments, to induce expansion of CMV-specific $CD8^+$ T cells. **(i, j)** Percentage of $hCD45^+$ cells, $CD3^+$ T cells and $CD8^+$ T cells in vaccinated mice at day 7 **(I)** and day 14 **(j)**. **(k-m)** *In vivo* engraftment and expansion of $hCD45^+$ cells **(k)**, $CD3^+$ T cells **(l)** and $CD8^+$ T cells **(m)** at day 7 and 14. **(d, e, i-m)** Data is shown as mean ± SEM (n=8). Statistical analyses were performed using repeated measures one-way ANOVA followed by Bonferroni correction comparing all pairs of columns **(d, e)**, an unpaired T-test **(i, j)** or a paired T-test **(k-m)**. **P<0.05, **P<0.01, ***P<0.001*.
