# Supplementary material for: Human CD34+-derived complete plasmacytoid and conventional dendritic cell vaccine effectively induces antigen-specific CD8+ T cell and NK cell responses in vitro and in vivo
Source: Cell Mol Life Sci. 2023 Sep 20;80(10):298. doi: 10.1007/s00018-023-04923-4 (PMC10511603; doi:10.1007/s00018-023-04923-4)
Supplement: Supplementary file 8 — Supplementary file8 (PDF 662 KB) [file 18_2023_4923_MOESM8_ESM.pdf]

## Supplementary figure 7

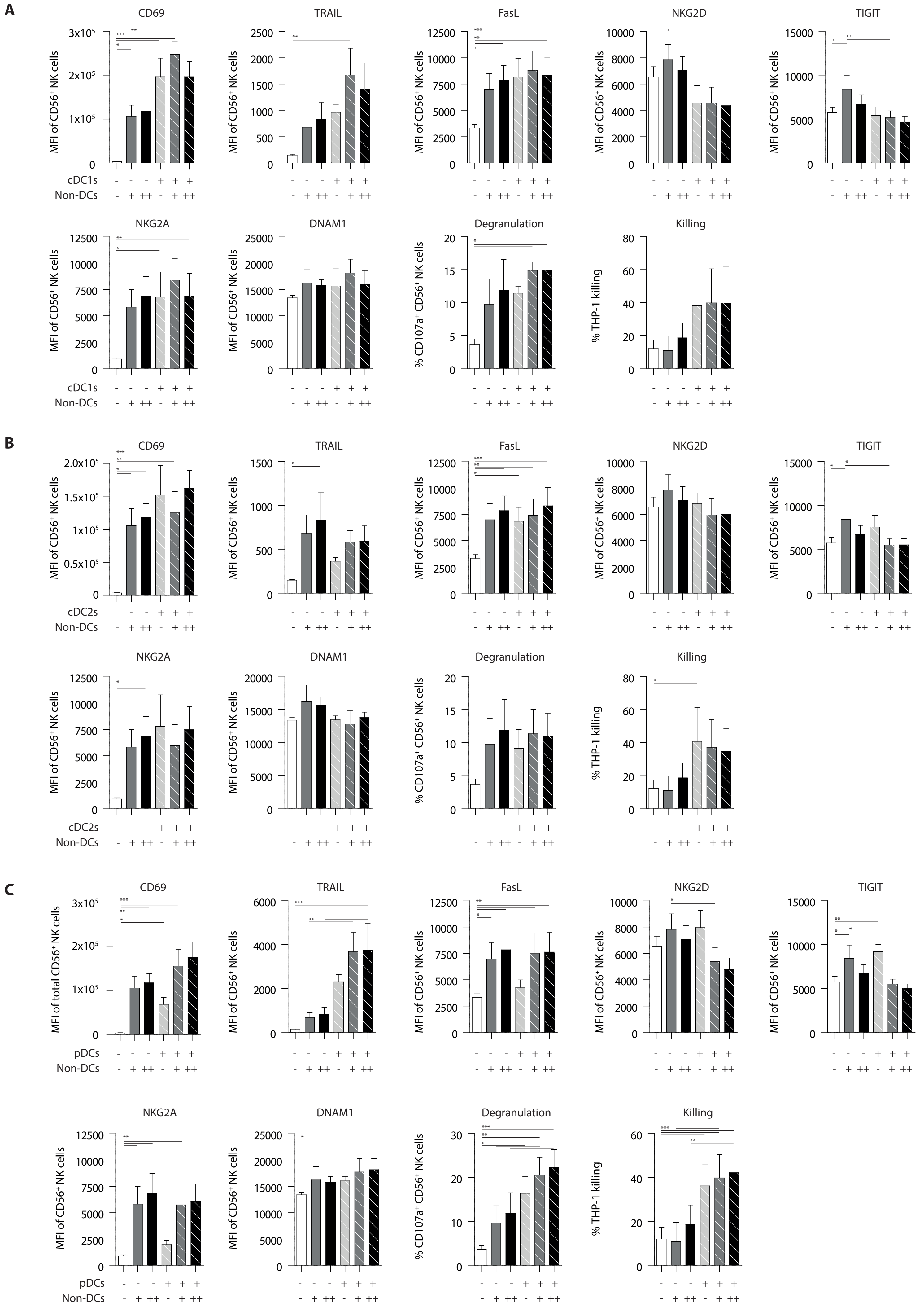

D

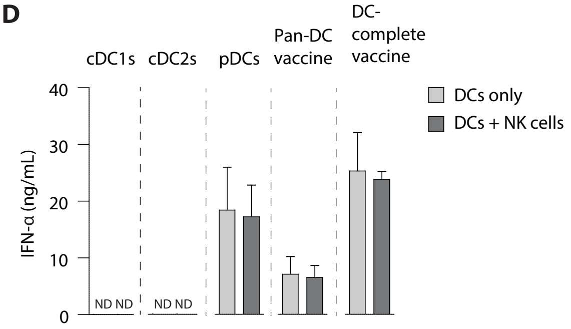


E

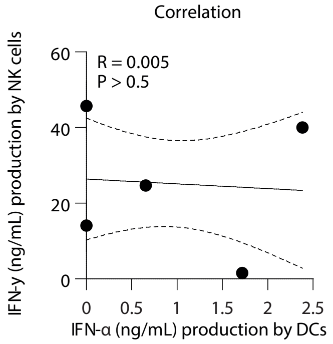


**Supplementary figure 7. cDC1, cDC2 and pDC-mediated NK cell activation is not hampered by non-DCs (a-c)** MFI of activating and inhibitory molecules, degranulation and THP-1 killing by NK cells after 2 days co-culture with cDC2s, cDC1s or pDCs in the absence or presence of non-DCs. **(d)** Release of pro-inflammatory cytokine IFN-α by 1 hour matured DCs in the absence or presence of NK cells. **(e)** Correlation of DC-derived IFN-α and NK cell-derived IFN-γ. **(a-e)** Data is shown as mean ± SEM (n=3). Statistical analyses were performed using a paired T-test **(e)**, a Pearson correlation **(e)** or repeated measures one-way ANOVA followed by Bonferroni correction comparing selected pairs of means **(a-c)**. **P<0.05, **P<0.01, ***P<0.001*.
