# Supplementary material for: Human CD34+-derived complete plasmacytoid and conventional dendritic cell vaccine effectively induces antigen-specific CD8+ T cell and NK cell responses in vitro and in vivo
Source: Cell Mol Life Sci. 2023 Sep 20;80(10):298. doi: 10.1007/s00018-023-04923-4 (PMC10511603; doi:10.1007/s00018-023-04923-4)
Supplement: Supplementary file 9 — Supplementary file9 (PDF 935 KB) [file 18_2023_4923_MOESM9_ESM.pdf]

Supplementary figure 8

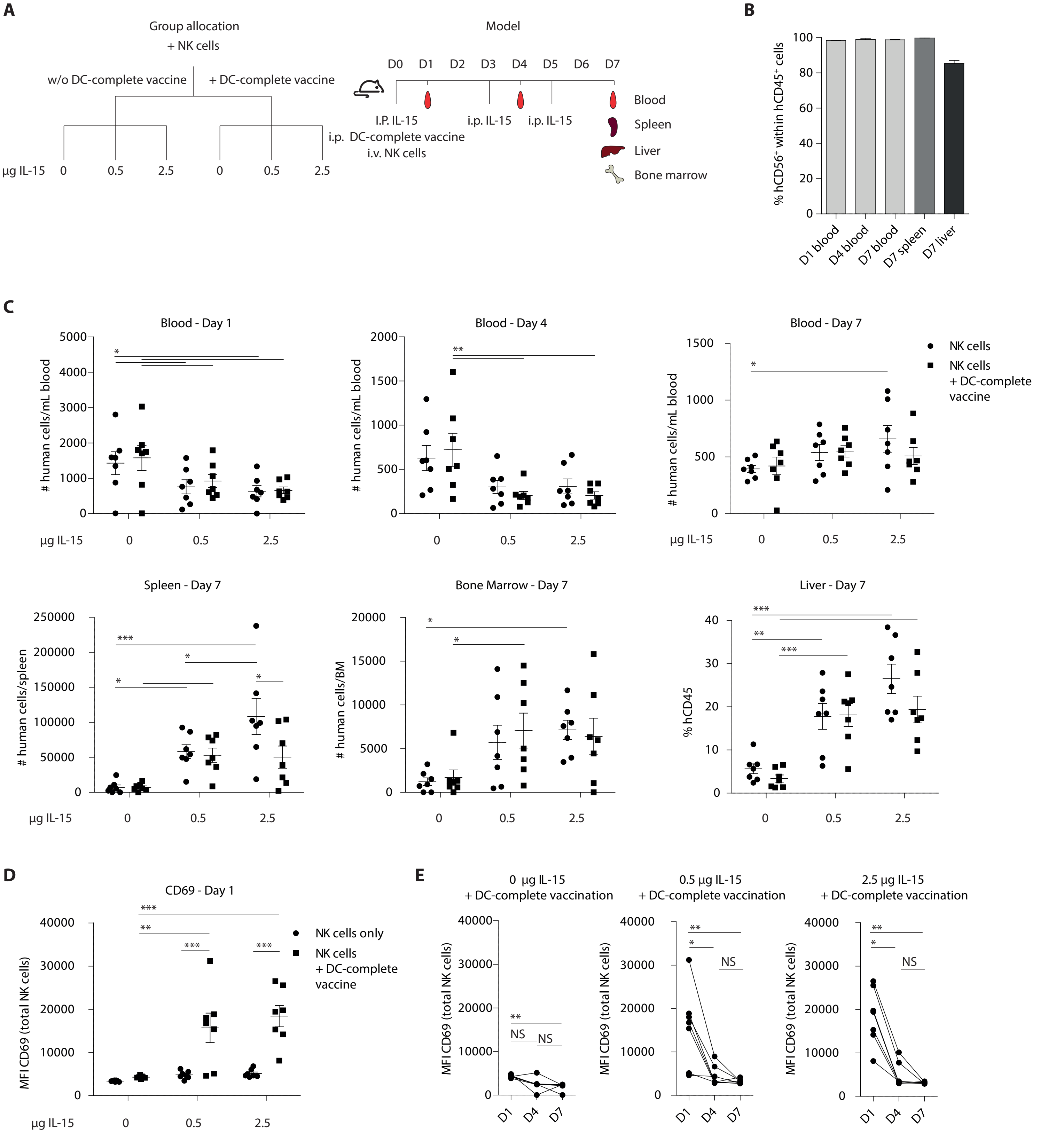

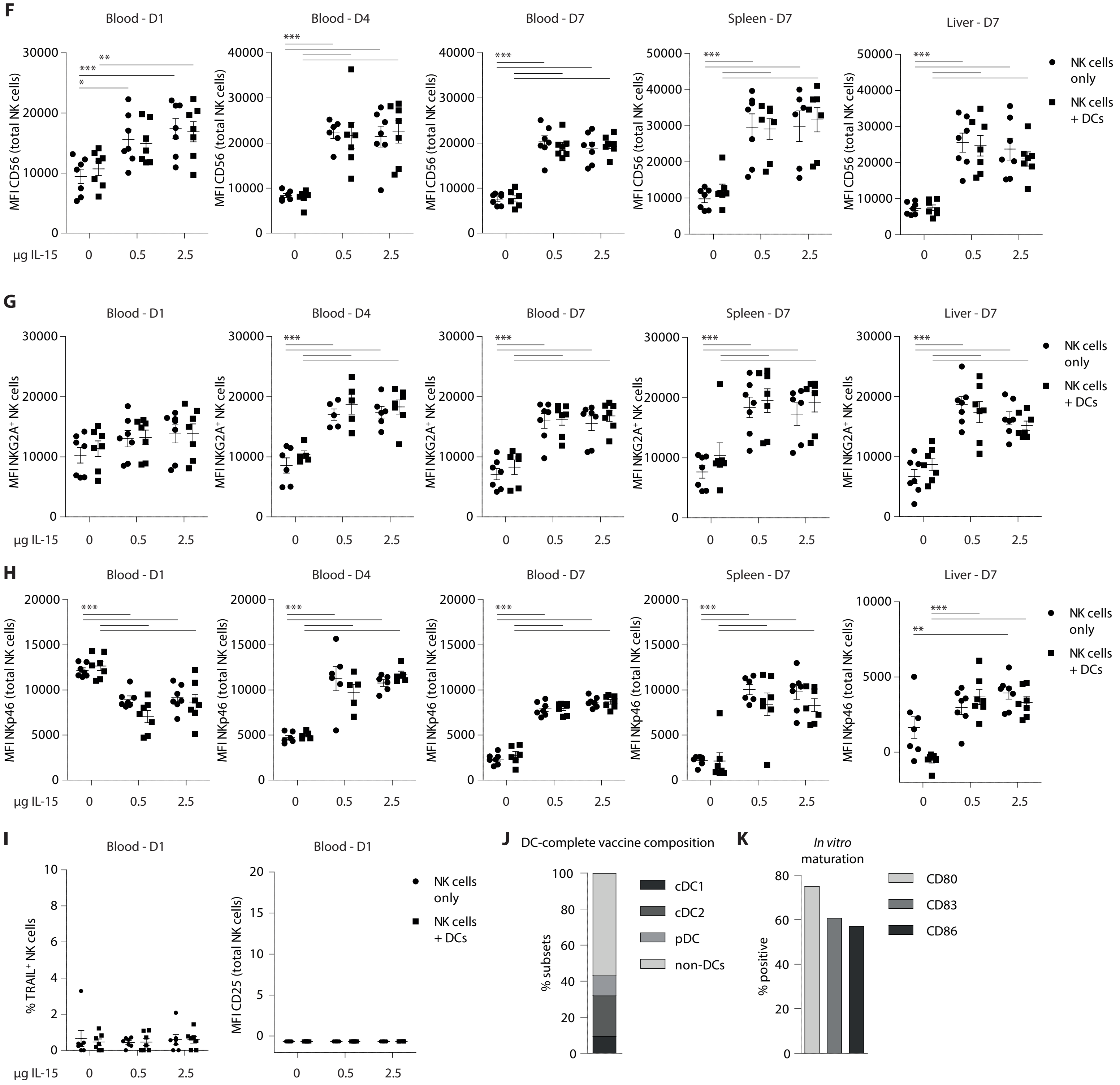

Supplementary figure 8. *In vivo* injected NK cells require IL-15 support. (a) Schematic overview of group allocation and experimental setup of the *in vivo* NK cell activation model (n =7 per group). **(b)** Percentage of CD56⁺ cells within hCD45⁺ cells from blood (day 1, 4 and 7) and spleen and liver (day 7). **(c)** Absolute numbers (blood, spleen, bone marrow) and frequency (liver) of hCD45⁺ cells in mice treated with or without DC-complete vaccination and supported with/without IL-15 at day 1, 4 (blood) and 7 (blood, spleen, bone marrow, liver). **(d, e)** MFI of CD69 on NK cells of mice treated with or without DC-complete vaccination and supported with/without IL-15 at day 1 **(d)** and followed over time **(e)**. **(f-h)** Expression of CD56, NKG2A and NKp46 on NK cells in mice vaccinated with or without DC-complete vaccination and supplemented with/without IL-15 support at day 1, 4 and 7 on NK cells isolated from blood, spleen and liver respectively. **(i)** Expression of TRAIL and CD25 on NK cells in mice vaccinated with or without DC-complete vaccination and supplemented with/without IL-15 at day 1. **(j)** Composition of the DC-complete vaccine used in this *in vivo* NK cell activation model. **(k)** Expression of co-stimulatory molecules CD80, CD83 and CD86 on the matured DC-complete vaccine before *in vivo* injection. Statistical analysis were performed using Two-Way ANOVA followed by Bonferroni correction comparing selected pairs of means **(c, d, f-h)** or one-way ANOVA followed by Dunn's correction comparing all pairs of means **(e).** *$P<0.05$, **$P<0.01$. ***$P<0.001$.*
