# Supplementary material for: Human CD34+-derived complete plasmacytoid and conventional dendritic cell vaccine effectively induces antigen-specific CD8+ T cell and NK cell responses in vitro and in vivo
Source: Cell Mol Life Sci. 2023 Sep 20;80(10):298. doi: 10.1007/s00018-023-04923-4 (PMC10511603; doi:10.1007/s00018-023-04923-4)
Supplement: Supplementary file 10 — Supplementary file10 (PDF 2780 KB) [file 18_2023_4923_MOESM10_ESM.pdf]

Supplementary figure 9

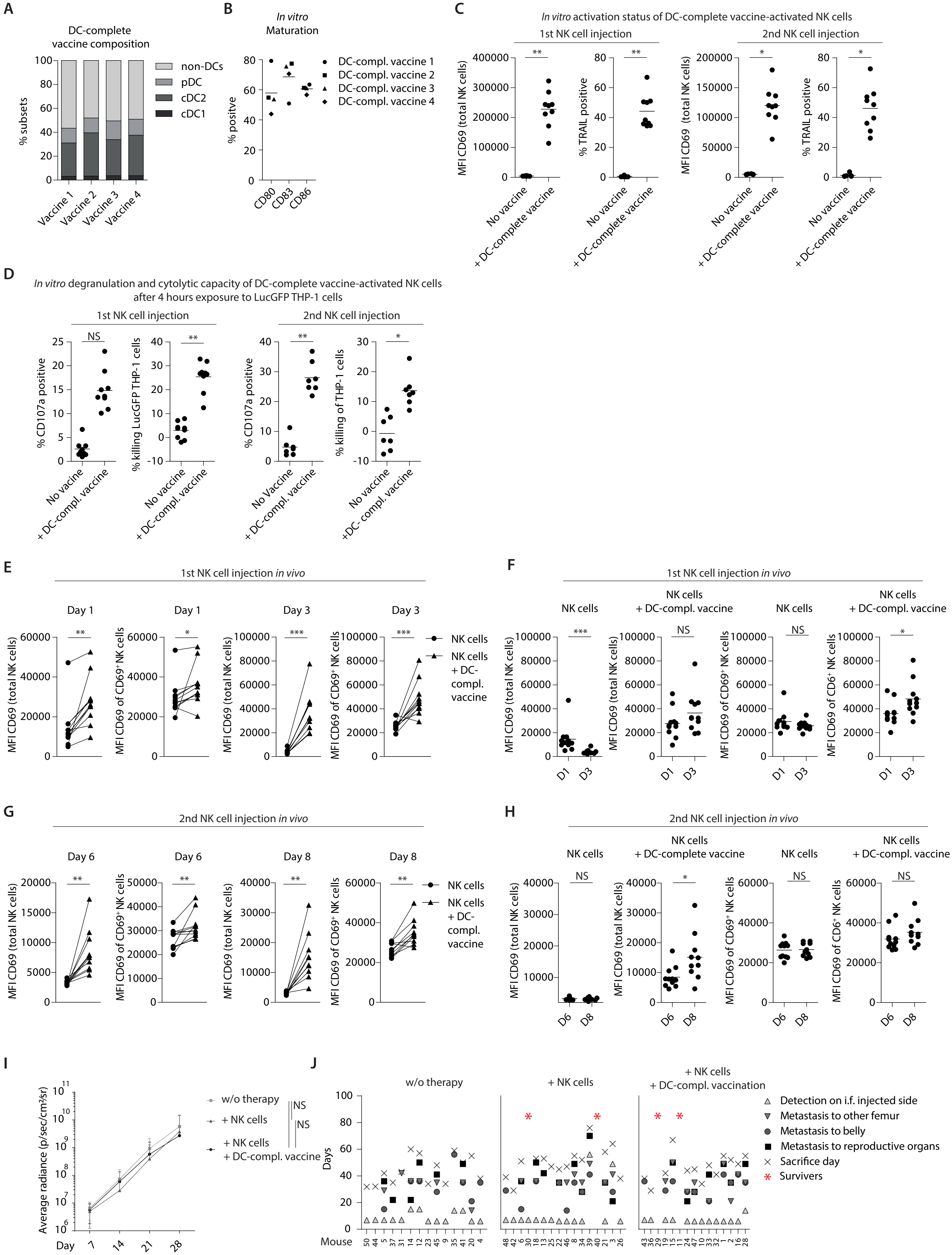

K

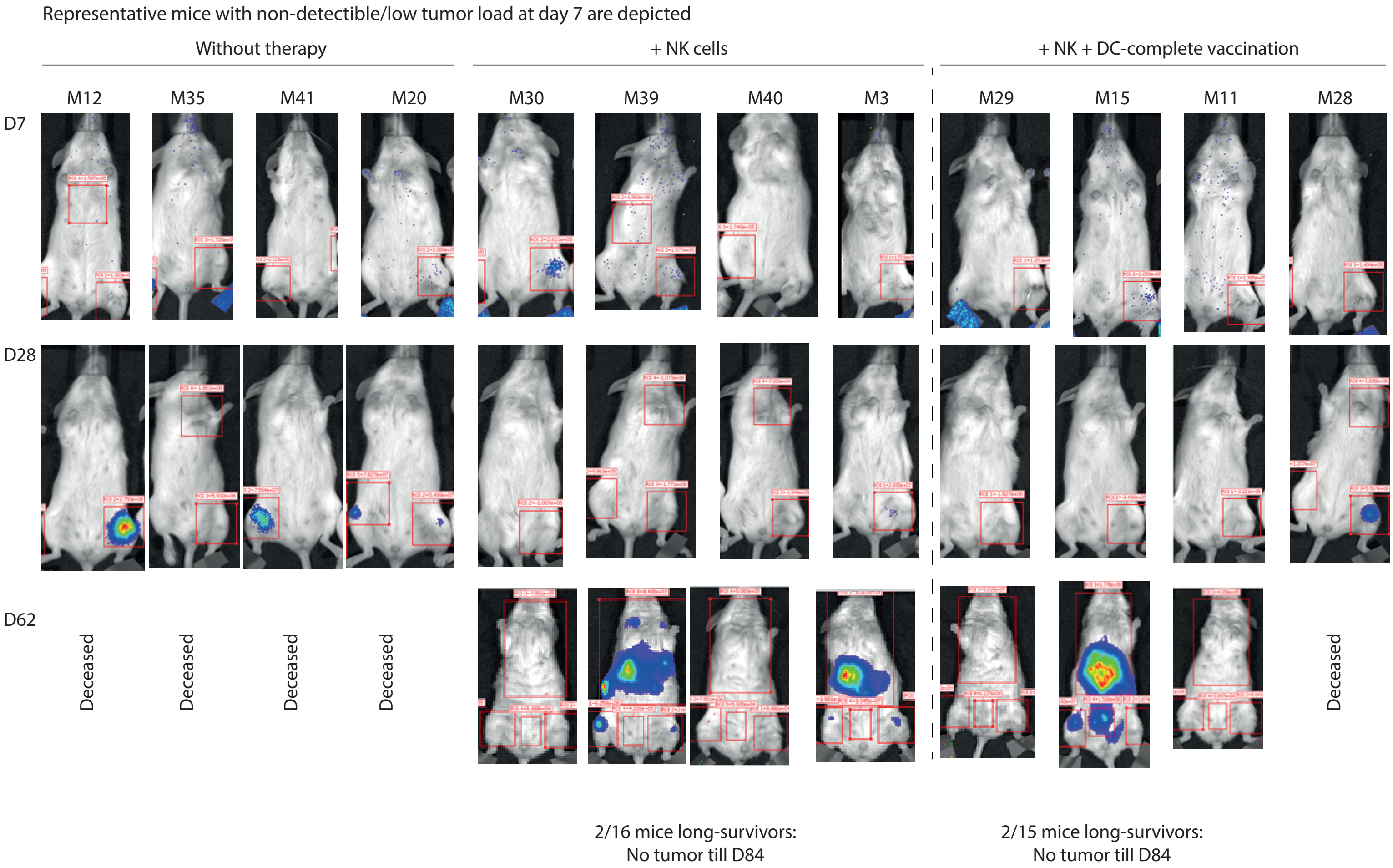

Supplementary figure 9. *Ex vivo* and *in vitro* evaluation of DC-complete vaccine potency and NK cell functionality. (a) Composition of DC-complete vaccine 1, 2, 3 and 4. **(b)** Expression of co-stimulatory molecules CD80, CD83 and CD86 on matured DC-complete vaccines before *in vivo* injection. **(c, d)** *Ex vivo* evaluation of expression of CD69 and TRAIL, degranulation and LucGFP THP-1 killing capacity of the NK cells used in the *in vivo* model. Each symbol represent one unique NK cell donor (n=9). **(e-h)** *Ex vivo* expression of CD69 on NK cells in blood from vaccinated mice after the 1st and 2nd vaccination **(e, f)** and 3rd and 4th vaccination **(g, h)**(n=11). **(i)** Radiance (photons/second/cm2/steradian) over time in LucGFP THP-1 bearing mice of different treatment groupd. **(j)** Overview showing time to detection of the LucGFP THP-1 tumor in the injected femur and the time to metastasis formation in the other femur, the belly region and reproductive organs using bioluminescence imaging (BLI) of each individual mouse. **(k)** Representative BLI images of mice with tumor progression over time. Statistical analyses were performed using non-parametric paired T-test **(c-h)** or mixed model two-way ANOVA followed by Tukey correction comparing selected pairs of means. **P<0.05, **P<0.01, ***P<0.001*.
